# A novel role for PGE_2_-EP_4_ in the developmental programming of the mouse ductus arteriosus: consequences for vessel maturation and function

**DOI:** 10.1152/ajpheart.00294.2023

**Published:** 2023-08-11

**Authors:** Michael T. Yarboro, Naoko Boatwright, Deanna C. Sekulich, Christopher W. Hooper, Ting Wong, Stanley D. Poole, Courtney D. Berger, Alexus J. Brown, Christopher S. Jetter, Jennifer M. S. Sucre, Elaine L. Shelton, Jeff Reese

**Affiliations:** ^1^Department of Cell and Developmental Biology, Vanderbilt University, Nashville, Tennessee, United States; ^2^Division of Neonatology, Department of Pediatrics, Vanderbilt University Medical Center, Nashville, Tennessee, United States; ^3^Department of Pharmacology, Vanderbilt University, Nashville, Tennessee, United States

**Keywords:** EP_4_ receptor, patent ductus arteriosus, pressurized vessel myography, prostaglandin signaling, vascular development

## Abstract

The ductus arteriosus (DA) is a vascular shunt that allows oxygenated blood to bypass the developing lungs in utero. Fetal DA patency requires vasodilatory signaling via the prostaglandin E_2_ (PGE_2_) receptor EP_4_. However, in humans and mice, disrupted PGE_2_-EP_4_ signaling in utero causes unexpected patency of the DA (PDA) after birth, suggesting another role for EP_4_ during development. We used EP_4_-knockout (KO) mice and acute versus chronic pharmacological approaches to investigate EP_4_ signaling in DA development and function. Expression analyses identified EP_4_ as the primary EP receptor in the DA from midgestation to term; inhibitor studies verified EP_4_ as the primary dilator during this period. Chronic antagonism recapitulated the EP_4_ KO phenotype and revealed a narrow developmental window when EP_4_ stimulation is required for postnatal DA closure. Myography studies indicate that despite reduced contractile properties, the EP_4_ KO DA maintains an intact oxygen response. In newborns, hyperoxia constricted the EP_4_ KO DA but survival was not improved, and permanent remodeling was disrupted. Vasomotion and increased nitric oxide (NO) sensitivity in the EP_4_ KO DA suggest incomplete DA development. Analysis of DA maturity markers confirmed a partially immature EP_4_ KO DA phenotype. Together, our data suggest that EP_4_ signaling in late gestation plays a key developmental role in establishing a functional term DA. When disrupted in EP_4_ KO mice, the postnatal DA exhibits signaling and contractile properties characteristic of an immature DA, including impairments in the first, muscular phase of DA closure, in addition to known abnormalities in the second permanent remodeling phase.

**NEW & NOTEWORTHY** EP_4_ is the primary EP receptor in the ductus arteriosus (DA) and is critical during late gestation for its development and eventual closure. The “paradoxical” patent DA (PDA) phenotype of EP_4_-knockout mice arises from a combination of impaired contractile potential, altered signaling properties, and a failure to remodel associated with an underdeveloped immature vessel. These findings provide new mechanistic insights into women who receive NSAIDs to treat preterm labor, whose infants have unexplained PDA.

## INTRODUCTION

The ductus arteriosus (DA) is a fetal vessel that shunts blood past the lungs in utero to protect the developing pulmonary vasculature and direct freshly oxygenated blood from the placenta into the systemic circulation. Although the DA is essential during fetal development, its postnatal closure is critical for circulatory transition to neonatal life. DA constriction is an elegant cascade of biological processes requiring acute changes in vascular tone, fluidity in cell phenotypes, and both prenatal and postnatal structural remodeling (1, 2). Frequently, disruptions in these genetic, environmental, and developmental processes result in failure of the DA to close, termed persistent patency of the DA (PDA). PDA comprises ∼10% of congenital heart defect cases in the United States (3) and is disproportionately common among preterm (64% at 27–28 wk) and very preterm infants (87% at 24 wk) (4). Adverse outcomes related to PDA can be severe, especially in preterm and low-birth weight neonates (5).

Prostaglandin signaling plays a critical role in the development and function of the DA. Prostaglandin precursors produced by the cyclooxygenase enzymes (COX)-1 and -2 are converted to prostaglandin E_2_ (PGE_2_) by specific PGE synthases. PGE_2_ actions are mediated by a family of G protein-coupled receptors, including the PGE receptors EP1, -2, -3, and -4, which have diverse cellular distribution and functions (6–9). PGE_2_ has potent vasodilatory effects on the DA and is clinically used to maintain DA patency after birth in newborns with cyanotic congenital heart lesions. Conversely, COX inhibitors are used to induce constriction of hemodynamically significant PDAs in preterm infants (5). EP_4_, encoded by *Ptger4*, is the primary prostanoid receptor expressed in both the rat (10) and human (11, 12) DA and is upregulated compared with surrounding vessels (13, 14). After birth, the initiation of respiration results in increased oxygen (O_2_) tension, and the initial constriction of the DA through mechanisms that are not fully understood (1, 2, 15–17). High levels of hydroxy prostaglandin dehydrogenase (HPGD) expression in the newly inflated lungs rapidly metabolizes circulating PGE_2_, removing a dilatory signal, and furthering constriction (18, 19).

The importance of developmental timing in fetal PGE_2_ signaling was first supported by observations that maternal exposure to COX inhibitors, given as a tocolytic to arrest preterm labor, results in fetal DA constriction after 30–32 wk of gestation, but not earlier in pregnancy (20, 21). In contrast, mothers who received COX inhibitors as tocolytics during late- but not midgestation had an increased risk of failed DA closure (PDA) in their offspring (22). This maturation-dependent response was confirmed pharmacologically in mice (23–25), and COX-1/COX-2 double KO mice were generated, which consistently produced a PDA phenotype coupled with congestive heart failure and early neonatal death (26, 27). In addition, three separate EP_4_ KO mouse models have been produced (28–30), all of which exhibit PDA and neonatal death with high penetrance, confirming the importance of the PGE_2_-EP_4_ receptor signaling axis in the DA. However, the COX double-KO and EP_4_ KO models are considered “paradoxical PDAs” because the removal of a vasodilatory signaling pathway would be expected to result in DA constriction rather than PDA (31). These findings suggest that PGE_2_-EP_4_ signaling may play a secondary developmental role in the DA, beyond acute regulation of DA tone. Although significant research has shown alterations in gene expression (32) and matrix biology of the EP_4_ KO DA (10, 33–36), a complete mechanistic explanation for their PDA phenotype has proven elusive.

Here, we used pharmacological inhibition studies, pressurized myography of isolated vessels, primary culture methods, and survival studies to assess the role of EP_4_ in DA development and function. Specifically, we set out to determine the developmental window in which EP_4_ is critical for proper DA development, and whether the EP_4_ KO DA resulted from impaired contractile potential, biomechanical properties, and/or deficient O_2_ response. We hypothesized that PGE_2_-EP_4_ signaling mediates a time-dependent developmental program essential for establishing the contractile properties and O_2_-sensing capabilities necessary to close the mature DA.

## METHODS

### Animals

Animal studies were conducted in accordance with approved standards of the Vanderbilt Institutional Animal Care and Use Committee and the National Institutes of Health. CD1 and C57BL/6J wild-type (WT) mice were acquired at reproductive age from The Jackson Laboratory (Bar Harbor, ME). *Ptger4* (+/−) mouse lines were established with permission from Drs. Breyer (30) and Narumiya (29) (CARD, Kumamoto, Japan) and are henceforth designated B6.129S6-*Ptger4^tm1^*^.2Matb^ and B6;129-*Ptger4*^tm1Sna^, respectively. Both models were maintained on a C57BL/6J background. The B6.129S6-*Ptger4^tm1^*^.2Matb^-null allele was crossed onto a CD1 background via 10 generation outcross as previously described (26, 37). KO animals were generated by timed mating of EP_4_ (+/−) breeders from 0700 to 1000 h with a vaginal plug designated as D1 (days postcopulation). All litters were delivered via cesarean section after maternal injection of 0.3 mL of 1.25% avertin (2,2,2-tribromoethanol in *tert*-amyl alcohol, Sigma-Aldrich) and brief isoflurane inhalation to ensure adequate fetal anesthesia, followed by cervical dislocation of the dam. In some studies, one uterine horn of anesthetized D17 pregnant females was briefly exteriorized to permit transuterine injection of selective receptor antagonists into the fetal peritoneal cavity. Dams were allowed to recover and ambulate for 30 min before euthanasia. For these experiments, drugs were tinted with Chicago blue B dye to document accurate fetal intraperitoneal administration at the time of necropsy. Comprehensive studies on sex differences in mouse and chick models of PDA (38, 39) do not show evidence of sexual dimorphism. Moreover, there are no sex differences in PDA incidence nor response to pharmacological treatment in preterm infants with PDA (40, 41); thus, no exclusions were made on the basis of sex, and male and female mice were included in all studies at their natural sex distribution, which have approximately equal values in our animal colony.

### Quantitative Real-Time RT-PCR

The DA and similarly sized ascending aorta (Ao) segments were collected from whole C57BL/6J litters and pooled by tissue with a minimum of three litters per time point (*D15*–*D19*). EP_4_ KO DA and Ao were collected individually and pooled later for three samples representing a minimum of five litters. RNA was extracted using TRIzol (Life Technologies) and bead homogenization (BeadMill24, Fischer Scientific), with cDNA generated using a SuperScript VILO cDNA Synthesis Kit (Invitrogen). RT-qPCR was performed using the StepOne Plus Real-Time PCR System and software, with TaqMan Fam-tagged primers (Supplemental Table S1:https://doi.org/10.6084/m9.figshare.23802282) and Vim-tagged 18S housekeeping standards (Applied Biosystems). Three technical replicates (ΔΔCT) were used. Fold change was calculated by setting D15 *Ptger1* expression (for time course), WT littermate expression (for genotype), D17 expression (for maturity markers), or DA/Ao expression (for strain) as indexed values to 1.

### Vessel Myography

Fetuses were delivered on D19 via terminal cesarean section, their thoracic cavities opened, and submerged in chilled and deoxygenated modified Kreb’s buffer, containing (in mM) 109 NaCl, 4.7 KCl, 2.5 CaCl_2_·2H_2_O, 0.9 MgSO_4_, 1.0 KH_2_PO_4_, 11.1 glucose, and 34 NaHCO_3_ (pH 7.3), as previously described (42). Fresh DA segments were excised, mounted in custom myography chambers (University of Vermont), and maintained at 37.5°C under deoxygenated conditions. Vasoreactivity of vessels was assessed via pressurized-vessel myography as previously described (43). Vessels were maintained at 5 mmHg for 40 min before being raised in 10 min, 5 mmHg steps to physiological pressure of 20 mmHg. Vessels were then subjected to two 10-min exposures to 50 mM KCl under deoxygenated constrictions to verify reactivity and integrity. Lumen diameter was recorded via inverted light microscope and video capture and analysis (IonOptix). The response to pharmacological agents or changes in gas concentrations were assessed by altering conditions in a 20 mL recirculating volume. Studies were performed on a minimum of six to eight vessels representing at least three litters. Findings are presented as percent change, percent reversal from a preconstricted baseline, or average lumen diameter.

### Inhibitor Studies

**Figure 1.**
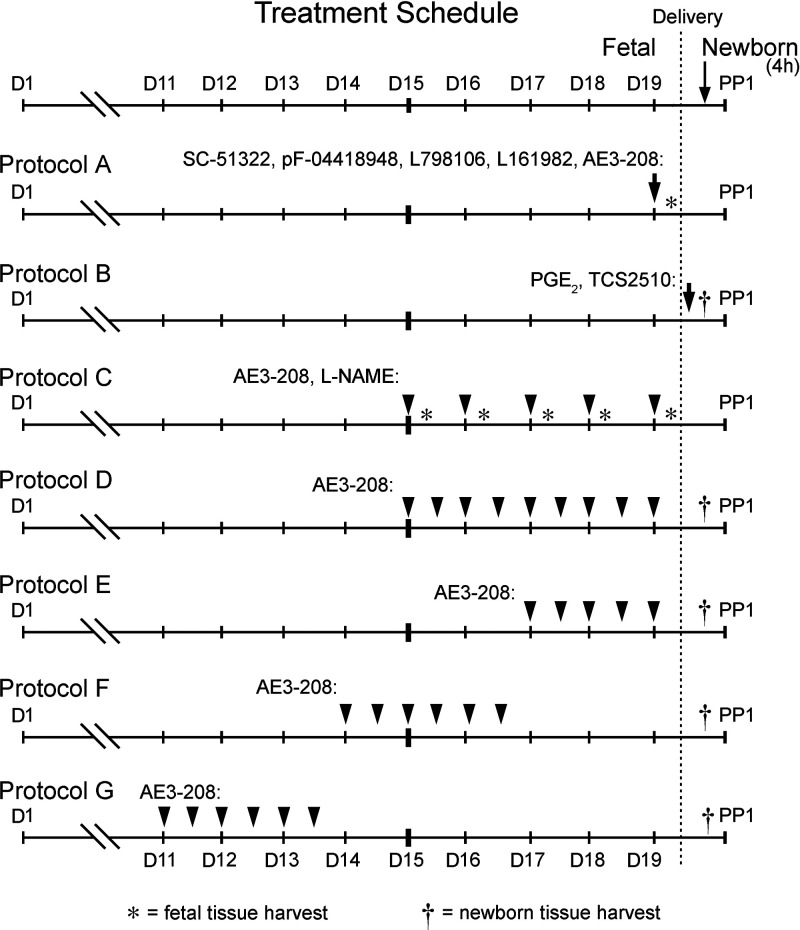
Drug treatment protocols. Pregnant dams were treated with selective antagonists to the prostaglandin E receptors EP_1_ (SC-51322), EP_2_ (pF-04418948), EP_3_ (L798,106), and EP_4_ (L161,982, ONO-AE3-208), a selective agonist to EP_4_ (TCS2510), prostaglandin E_2_ (PGE_2_), or an inhibitor of nitric oxide (NO) synthase *N*^G^-nitro-l-arginine methyl ester (l-NAME) on the indicated days of gestation [*day 1* (*D1*) = presence of vaginal plug]. Fetal exposure studies: *Protocol A* examined the effect of acute EP receptor antagonism on the in utero ductus arteriosus (DA), with DA scoring 4 h after maternal intraperitoneal administration. Neonatal injection studies: *Protocol B* examined the effect of postnatal EP receptor stimulation on DA constriction, with drugs being administered to offspring 30 min after delivery and DA scoring 4 h later. Maternal gavage studies: *Protocol C* examined how inhibition of EP_4_ or NO synthase affected fetal DA patency on select days of pregnancy. Drugs were administered the morning of the indicated day and DA patency assessed 4 h later. Chronic gavage studies: *Protocol D* examined the effect of chronically inhibiting EP_4_ signaling over a defined window of pregnancy. Mice were delivered via cesarean section on the morning of *D19* with their DAs scored 4 h later. This approach was repeated during discrete windows in *Protocols E, F, and G*. PP1, *postpartum day 1*.

Drugs and compounds were administered to pregnant dams or pups at different gestational time points and with varying methodologies (Fig. 1). *Protocols A* and *C* addressed acute drug effects on the fetal DA, *protocol B* addressed acute effects on the neonatal DA, and *protocols D–G* addressed chronic effects on the fetal DA. In *protocol A*, selective antagonists of the EP receptors EP_1_ [SC-51322, 10 mg/kg/dose once (44, 45); ip; TOCRIS], EP_2_ [PF-04418948, 10 mg/kg/dose once; ip (46); TOCRIS], EP_3_ [L-798,106, 5 mg/kg/dose once (47); ip; TOCRIS], and EP_4_ [L-161,982, 100 mg/kg/dose once hourly, 4 total (48) (originally 10 mg/kg/dose once, one total, but increased to 4 total doses because of short in vivo half-life); ip; TOCRIS; or AE3-208, 10 mg/kg/dose once; ip; ONO Pharmaceuticals] (49, 50) were administered to CD1 WT dams at 0800 h on the morning of *D19*. Four hours later, pups were delivered via cesarean section, their chests opened, and their DAs visually scored for percent patency by a single observer (M.T.Y. or N.B.) blinded to treatment group, using a five-point noncontinuous scale (0, 25, 50, 75, and 100%) comparing the DA to the main pulmonary artery as previously described (1, 24, 26, 51–53). Visual scoring renders immediate determination of DA patency and corresponds to measurements from histological scoring (24, 25). Dosage and timing of AE3-208 administration was based on previously published optimization studies (50). The 4-h incubation time between drug administration and DA scoring was based on previous COX inhibitor and EP_4_ receptor antagonist studies in pregnant rodents (23, 50, 54) and the 3–6 h period of time required for normal DA constriction after birth (55). For *protocol B*, CD1 WT litters were delivered at 0800 h on the morning of *D19*, resuscitated, and maintained on a 37.5°C warming pad. At 30 min after birth, pups were administered PGE_2_ (10 µg/kg/dose once hourly, 4 total; 13 µL ip; TOCRIS) or an EP_4_-selective agonist (TCS2510, 10 µg/kg/dose once hourly, 4 total; 13 µL ip; TOCRIS). After 4 h, pups were euthanized via isoflurane inhalation and their DAs scored. Terminal isoflurane exposure was considered to have less effect on vessel diameter than alternative euthanasia methods. In addition, pups in control and treatment groups were treated similarly, minimizing the effect of anesthetic choice. *Protocol C* used C57BL/6J dams administered either an EP_4_-selective antagonist (AE3-208, 10 mg/kg/dose once; 0.2 mL gavage) or a nitric oxide (NO) synthase inhibitor *N*^G^-nitro-l-arginine methyl ester (l-NAME, 180 mg/kg/dose once; 0.2 mL gavage; Cayman) at 0800 h on the morning of a day of pregnancy (*D15*–*D19*). At 4 h after gavage, pups were delivered via cesarean section and their DAs scored. For *protocol D*, C57BL/6J dams were administered a selective EP_4_ antagonist (AE3-208, 100 mg/kg/dose twice daily; 0.2 mL gavage) at 0800 and 2000 h during discrete windows of pregnancy (*D*: *D15*–*D19*, *E*: *D17*–*D19*, *F*: *D14*–*D16*, *G*: *D11*–*D13*). For *protocols D* and *E*, a final dose was administered at 0800 h on *D19*. All litters were delivered and their DAs were scored at 1200 h. Pups were delivered via cesarean section, resuscitated, and placed on a warming pad heated to 37.5°C. After 4 h, pups were euthanized and their DAs were scored. Those who died in less than 2 h after birth were excluded from further analysis.

### Cultured Primary Cells

Anesthetized CD1 WT pups were delivered via cesarean section and their DA and Ao tissues quickly explanted via dissection. Breathing pups were excluded and euthanized humanely. Explants were submerged in Dulbecco’s modified Eagle’s medium (Gibco) supplemented with 10% FBS (Atlanta Biologicals) and 2% penicillin-streptomycin (Gibco) warmed to 37.5°C. Explants were further cleaned to remove the endothelium and adventitial layer and cut along their length. These medial layer explants were then transferred to a sterile 12-well culture plate (Denville) with fresh, warmed, media and gently pressed into the plastic of the well. Three explants were cultured per well in copper-lined incubators (HeraCell) at 37.5°C and normoxia with 5% CO_2_ and frequent media changes (every other day). Explants were allowed to grow until a wide corona of cells was present. Explants were then removed from the well using an aspirating pipette, the cells were rinsed three times with cold PBS (Corning), and lifted with 0.25% trypsin (Gibco). For wound healing assays, P2 cells were seeded into a new 12-well plate at 500 cells per well and allowed to grow until a uniform monolayer was formed. Cells were then serum starved for 24 h and scratched with a 200-µL pipette tip, rinsed twice with serum-free media, and given media supplemented with an EP_4_-selective antagonist (AE3-208, 10uM) (49) or DMSO (3 µL/mL; Sigma-Aldrich) vehicle control. DMSO concentration was minimized (≤0.3%) to limit adverse effects (56, 57). Images were taken immediately following (T1) and 24 h after wound creation (T2). Images were analyzed with NIH ImageJ software to determine wound closure. For immunohistochemistry, P1 cells were seeded into a four-well glass-bottomed chamber slide (Lab-Tek II; Thermo Fisher Scientific) at 1,000 cells per well and allowed to attach for 24 h. Cells were then rinsed with PBS, fixed with 4% PFA (Fisher) for 15 min, and rinsed with PBS for 45 min. Cells were blocked for 1 h at room temperature [90 mL PBS, 10 mL goat serum (R&D Systems), 100 µL Triton X (Thermo Fisher Scientific)]. Cells were incubated in Caldesmon (1:100; Abcam) and α-smooth muscle actin (α-SMA; 1:250; Sigma) primary antibodies in goat-blocking serum overnight at 4°C. Cells were washed for an hour with PBS, then incubated in GαR Alexa Fluor 488 (1:2,000, green; Abcam) and GαM Alexa Fluor 568 (1:2,000, red; Abcam) secondary antibodies diluted in goat-blocking serum with DAPI (0.1 µg/mL; Thermo Fisher Scientific) for 3 h at room temperature. Cells were then washed with PBS for 30 min, cover-slipped with aqueous mounting media, and imaged with an epifluorescence microscope.

### RNA In Situ Hybridization

Anesthetized CD1 WT pups were delivered via cesarean section, their thoracic cavities opened, and their bodies submerged in cold deoxygenated Kreb’s buffer. Whole outflow tracts were extracted, fixed in 10% phosphate-buffered formalin (Fisher) for 30 min, dehydrated in a four-point ethanol series (Fisher), cleared for 5 min with xylenes (Fisher), and embedded in paraffin blocks. Microtome sections (∼8 µm) were obtained (Thermo Fisher Scientific, Shandon Finesse 325) and floated onto slides.

Fixed paraffin slides containing 8-µm tissue sections from D19 CD1 WT outflow tracts were pretreated according to ACD’s standard RNAscope protocol (Pretreatment Kit 4, ACD). Target probes against transcripts of mouse *Ptger4* (Mm-Ptger4-C3 *Mus musculus*, transcript variant 1, Cat. No. 441461-C3, ACD) and kit-based (RNAscope Multiplex Fluorescent Reagent Kit v2, ACD) preamplifier, amplifier, and fluorescent-labeled probes (Atto 550, Orange, ACD) were subsequently hybridized, and results were visualized via epifluorescence microscopy. Traditional RNA in situ hybridization using ^35^S-labeled sense and antisense probes for *Ptger1*, *Ptger3*, and *Ptger4* was performed as previously described (26).

### Hyperoxia Studies

Litters produced by EP_4_ (+/−) intermatings were delivered via cesarean section, resuscitated, and placed inside a mouse isolation chamber supplemented with humidified medical grade O_2_ to a concentration of ∼70% O_2_. Pups were euthanized via isoflurane inhalation after 4 h and their DAs were scored. For 24- and 48-h studies, pups were delivered as described, then fostered to a CD1 WT dam, and maintained in hyperoxia chambers for up to 2 days. Pups that died in less than 2 h after birth were excluded from further analysis.

### Statistics

For all studies, a *P* value < 0.05 was considered significant. For clarity, data are presented as means ± SE. Drug doses represent the cumulative final molar concentration in the recirculating system. Myography data were considered as percent change from baseline, percent reversal from preconstricted baseline, or change in lumen diameter. Normality of data was assessed by either Shapiro–Wilk or Kolmogorov–Smirnov tests, and ANOVA results were considered valid for datasets with consistent variance. Single-point comparisons were analyzed using unpaired Student’s *t* test. Multipoint concentration-response curves were compared using repeated measures two-way ANOVA with post hoc Bonferroni multiple-comparison test when significance was detected, and in some cases, nonlinear curve fitting was modeled (GraphPad Prism 6). Visually scored DA data and premature death data are represented as frequency distributions and analyzed by χ^2^ test. RT-qPCR data were analyzed between and among groups by Kruskal–Wallis test. Wound healing and myogenic tone data were analyzed by paired Student’s *t* test comparing initial and final points of the same sample.

## RESULTS

### EP_4_ is the Primary Mediator of Prostanoid Signaling in the DA

**Figure 2.**
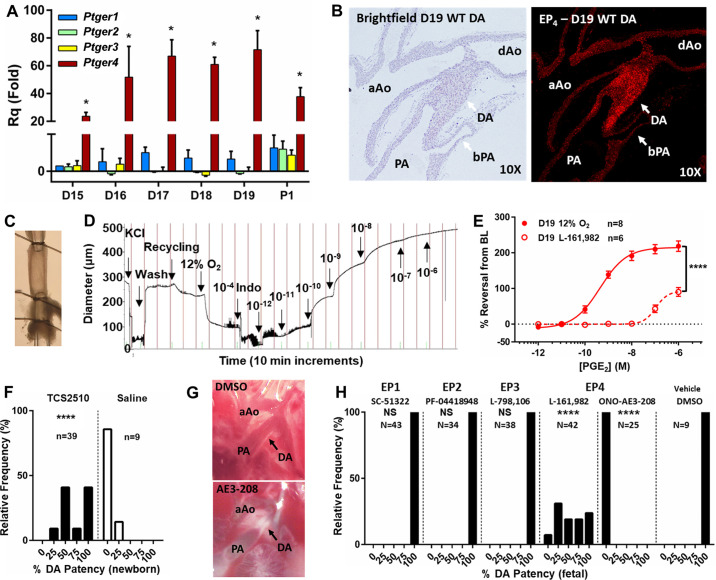
Prostaglandin E_2_ (PGE_2_) receptor EP_4_ is the predominant EP receptor in the mouse ductus arteriosus (DA). *A*: time course of EP receptor expression in the DA. *Ptger4* expression increased with advancing gestation compared with *day 15* (*D15*) (*P* < 0.05; Kruskal–Wallis) and was significantly greater than individual EP subtypes at each gestational stage (*P < 0.05; Kruskal–Wallis) (*n* = 3 biological replicates). *B*: localization of *Ptger4* (EP_4_) expression in the DA and outflow tracts. *C*: cannulated ex vivo preparation of *D19* CD1 wild-type (WT) DA for vessel myography. *D* and *E*: representative tracing of a PGE_2_ concentration response curve (CRC; *D*) and cumulative response curves of EP_4_-inhibited and control DAs following O_2_-induced preconstriction (*E*), demonstrating the potent and EP_4_-predominant effects of PGE_2_ on the isolated DA. *F*: in vivo studies demonstrate a shift in neonatal DA patency rates in response to injections of the selective EP_4_ agonist TCS2510, resulting in patent DA (PDA). *G*: representative images of DA patency in response to selective EP receptor antagonists, scored on a 5-point noncontinuous scale, showing 100% patency (*top*) and 0% patency (*bottom*). *H*: in utero exposure to selective EP receptor antagonists resulted in fetal DA constriction in response to 2 selective EP_4_ antagonists, but not to EP_1_ EP_2_, or EP_3_ antagonists. AE3-208 was used in subsequent EP_4_ inhibitor studies because of its increased potency and DA effects. ****P < 0.001 compared with control (*E*) or vehicle (*F* and *H*) (*A*, Kruskal–Wallis; *E*, two-way ANOVA; *F* and *H*, χ^2^). aAo, ascending aorta; BL, baseline; bPA, branch pulmonary artery; dAo, descending aorta; NS, not significant; PA, pulmonary artery.

To determine the magnitude and timing of PGE receptor expression, RT-qPCR was performed on *D15*–*D19* DAs for the four prostanoid receptor genes, *Ptger1*, *Ptger2*, *Ptger3*, and *Ptger4* (EP1, -2, -3, and -4, respectively) (Fig. 2*A*). EP_4_ was significantly upregulated at *D16* and maintained this upregulation into the postnatal period. Cell-specific localization of *Ptger4* transcripts revealed low levels of EP_4_ expression in the *D19* pulmonary artery and Ao but strong expression in the medial layer of the DA (Fig. 2*B*). *Ptger4* expression persisted in the medial and endothelial layers of the closed DA on *postpartum day 1* (Supplemental Fig. S1). Because EP_4_-null mice unexpectedly have a PDA phenotype, the role of EP_4_ in mediating acute DA tone was assessed through pressurized vessel myography. Isolated WT vessels (Fig. 2*C*) exposed to increasing concentrations of PGE_2_ (Fig. 2*D*) or the selective EP_4_ agonist TCS2510 (Supplemental Fig. S2*A*) displayed a potent concentration-dependent vasodilatory response. This effect was significantly attenuated by pretreatment with a selective EP_4_ antagonist (L-161,982) (Fig. 2*E*). Direct injection of L-161,982 into the fetal intraperitoneal compartment (Supplemental Fig. S2, *B–E*) also caused DA constriction, suggesting that EP_4_ mediates PGE_2_’s acute vasodilatory effects in the fetal DA. To determine the role of EP_4_ on DA tone in vivo, the EP_4_ agonist TCS2510 was administered to newborn pups following delivery (*protocol B*). After 4 h, DA closure was impaired in treated animals resulting in PDA (Fig. 2*F*), similar to the effects of PGE_2_ infusion in newborn infants. To determine the in vivo contributions of other EP receptors, DA patency was scored in fetuses 4 h after maternal injection with a selective EP receptor antagonist (*protocol A*). Although antagonism of EP1, -2, and -3 had no detectable effect on fetal DA caliber, antagonism of EP_4_ led to significant constriction of the DA (Fig. 2, *G* and *H*). Constriction of the fetal DA by AE3-208 was more extensive than by L-161,982; thus, AE3-208 was used for all subsequent EP_4_ antagonist experiments. Collectively, these data suggest that EP_4_ is the primary prostanoid receptor expressed in the DA and that it mediates the vasodilatory effects of PGE_2_.

### The Acute Role of EP_4_ in the DA is Dependent on Developmental Timing

**Figure 3.**
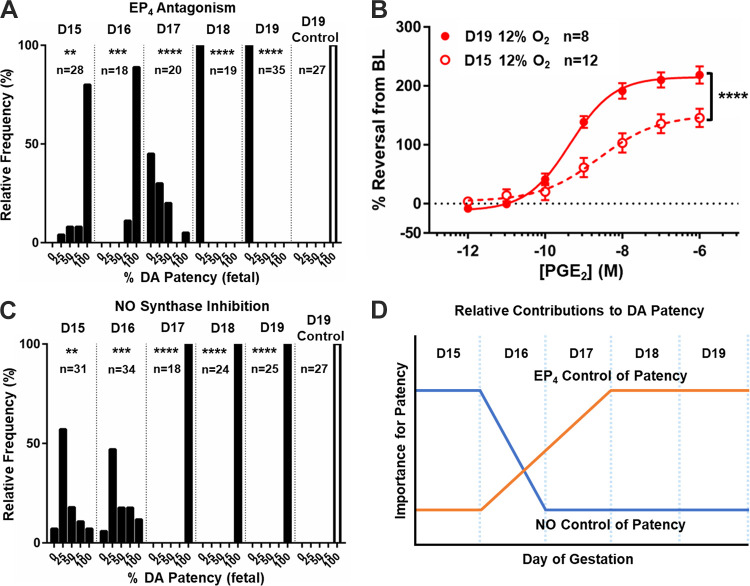
The role of prostaglandin E_2_ (PGE_2_) receptor EP_4_ in ductus arteriosus (DA) patency is dependent on gestational timing. *A*: response of the in vivo fetal DA to a selective EP_4_ antagonist demonstrates late DA constriction and a gestational stage-specific shift in sensitivity of the developing DA to disruption of PGE_2_-EP_4_ signaling between *days 16–17* (*D16*–*D17*) of pregnancy. *B*: cumulative PGE_2_ response curves similarly show that the preconstricted ex vivo immature *D15* DA is significantly less sensitive to PGE_2_ exposure compared with *D19* DAs. *C*: in contrast, the response of the in vivo fetal DA to a selective nitric oxide (NO) synthase inhibitor showed limited effects in late gestation but significant fetal DA constriction at immature time points. *D*: schematic diagram depicting reciprocal shifts in DA dependence on PGE_2_-EP_4_ and NO signaling for patency with advancing gestation. **P < 0.01; ***P < 0.005; ****P < 0.001 compared with controls (*A* and *C*) or between gestations (*B*) (*B*, two-way ANOVA).

To determine when EP_4_ gains a significant role as a vasodilator in the developing DA, dams were given a single dose of the AE3-208 selective EP_4_ antagonist on different days of pregnancy (*protocol C*). Antagonism of EP_4_ was found to have little effect on *D15* and *D16*, more significant effects on *D17*, and complete in utero DA constriction on *D18* and *D19* (Fig. 3*A*). Corresponding myography studies comparing PGE_2_ concentration-response curves (CRCs) in the term (*D19*) and premature (*D15*) DA revealed a significantly diminished response in the premature DA (Fig. 3*B*). In a parallel experiment, an NO synthase inhibitor (l-NAME) given to dams on different days of pregnancy revealed significant effects on DA tone on *D15* and *D16*, but not on *D17*–*D19* of gestation (Fig. 3*C*). Together, these data suggest that NO is the primary dilator in the fetal DA until *D17* when PGE_2_ acting via EP_4_ begins to subsume this role (Fig. 3*D*).

### EP_4_ is Indispensable for DA Development from *D17* to *D19*

Chronic pharmacological inhibition of the EP_4_ receptor during discrete windows of pregnancy was used to mimic the EP_4_ KO phenotype to determine when EP_4_ signaling serves in a developmental role. First, we observed that dams given the selective EP_4_ antagonist AE3-208 from *D15* to *D19* of pregnancy (*protocol D*) had pups with varying degrees of PDA at 4 h of age compared with fully closed DAs in vehicle-treated mice (Supplemental Fig. S3). The PDA phenotype in offspring exposed to chronic pharmacological inhibition was more pronounced in C57BL/6J than CD1 mice or mice with a mixed genetic background (Supplemental Fig. S3, *A* and *B*). Similarly, attempts to cross the EP_4_-null allele (B6.129S6-*Ptger4^tm1^*^.2Matb^) into CD1 mice resulted in increased survival of KO mice at weaning (Supplemental Fig. S3*C*) and loss of the PDA phenotype (Supplemental Fig. S3*D*). Although EP_4_ KO (B6;129-*Ptger4*^tm1Sna^) mice were born at the expected Mendelian ratio (20 KO, 24 WT, 47 Het), at weaning both EP_4_ KO models on either a C57BL/6J (4-KO, 142 WT, 188 Het) or CD1 (50-KO, 118 WT, 197 Het) background were no longer Mendelian, consistent with neonatal death. Although postneonatal losses of KO mice on the CD1 background still occurred, they were significantly less severe. These findings may be related to strain-specific differences in EP receptor expression (Supplemental Fig. S3*E*).

**Figure 4.**
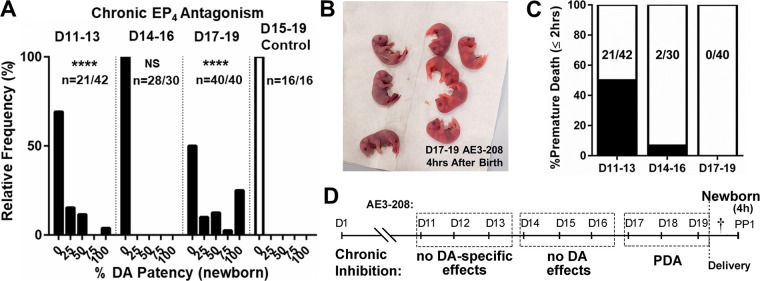
Prostaglandin E_2_ (PGE_2_) receptor EP_4_ is critical during the *day 17–19* (*D17*–*D19*) window for proper postnatal ductus arteriosus (DA) function. *A*: response of the neonatal DA to chronic EP_4_ antagonist exposure during discrete gestational windows demonstrating increased rates of failed DA closure at 4 h of age when drug was given over the late- but not midgestational window (*n* values represent animals surviving to 2 h after birth/total animals assessed). NS, not significant. *B*: representative image of a litter of chronically EP_4_ inhibited offspring (*D17*–*D19*), some of whom exhibited a dusky appearance frequently associated with patent DA (PDA). *C*: chronic exposure to the EP_4_ antagonist during *D11*–*D13* of gestation resulted in significant postnatal mortality associated with apnea, the need for constant stimulation, and early demise, suggesting that increased PDA rates among some *D11*–*D13* survivors (*n* values represent animals surviving to 2 h after birth/total animals assessed) (*A*) were related to nonspecific effects unrelated to DA status. Thus, while acute exposure to an EP_4_ antagonist (AE3-208) resulted in fetal DA constriction (Figs. 2*H* and 3*A*), prolonged exposure to the same drug produced PDA in newborns, but only in the late-gestational window (*D*) (*n* values superimposed on columns). ****P < 0.001 compared with control (*A*, χ^2^). †, time of tissue harvest; PP1, *postpartum day 1*.

We next compared chronic pharmacological EP_4_ inhibition during three shorter windows (*D11*–*D13*, *D14*–*D16*, and *D17*–*D19*) (*protocols E–G*), revealing that drug treatment over the *D14*–*D16* window had no effect on postnatal DA closure at 4 h of age, whereas inhibition from *D17* to *D19* resulted in a significant DA patency compared with control and a phenotype that recapitulated the EP_4_ KO (Fig. 4*A*). Pups similarly grew weak and dusky after birth, occasionally succumbing near 4 h of age (Fig. 4*B*). Inhibition during the *D11*–*D13* window revealed an EP_4_-dependent neonatal death phenotype independent of PDA, likely representing drug effects that are off-target or not specific to DA function (Fig. 4*C*). Together, these data show that EP_4_ plays a temporal role in DA development during the *D17*–*D19* window, which is indispensable for accomplishing postnatal DA closure (Fig. 4*D*).

### EP_4_ Plays a Non-matrix Role in Migratory Potential of DA VSMCs

**Figure 5.**
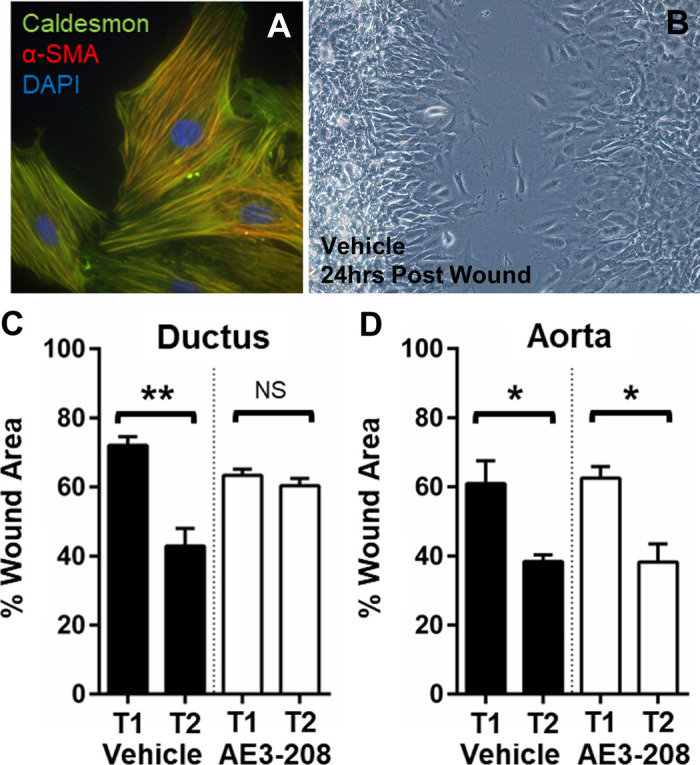
Prostaglandin E_2_ (PGE_2_) receptor EP_4_ antagonism disrupts cell migration in ductus arteriosus (DA) smooth muscle cells (SMCs) but not aortic (AO) SMCs. *A*: primary cultured P3 DA SMCs labeled with markers for mature vascular SMCs (VSMCs) demonstrated typical SMC characteristics after low passage. *B*: scratch assay studies demonstrated intact cell migration in a field of DA SMCs 24-h postwounding. *C* and *D*: cumulative results representing the response of vehicle-treated vs. EP_4_-selective antagonist-treated ductus (*C*) and AO SMCs to wound healing (*D*), suggesting that DA SMC, but not AO SMC, migration is dependent on EP_4_ signaling in vitro. NS, not significant; T1, 0-h postwound; T2, 24-h postwound; %wound area determined as average percentage of 3 visual fields with 3 technical replicates and 3 biological replicates each. *P < 0.05, **P < 0.01 compared with T1 baseline. (*C* and *D*, *t* test).

EP_4_ signaling contributes to cell migration and invasion in various tissues (58–60). Specifically, EP_4_-activated EPAC1 has been shown to promote smooth muscle cell (SMC) migration independent of matrix or proliferation in the rat DA (33). To determine if EP_4_ plays a role in the vascular smooth muscle cell (VSMC) migration that leads to DA closure (61–64) in the mouse, we used a primary culture model. Low-passage VSMCs were cultured from explants of term mouse DA and Ao, shown to express mature muscle markers in >99% of cells (Fig. 5*A*) and subjected to treated scratch assays (Fig. 5*B*). DA cells that were wounded and incubated for 24 h migrated faster than Ao cells in the scratch assay. EP_4_ antagonism with AE3-208 had a significant negative effect on wound healing and cell migration rates in DA but not Ao cells, suggesting that EP_4_ does contribute to the migratory potential of DA VSMCs and that this effect is vessel specific (Fig. 5, *C* and *D*).

### Deletion of EP_4_ Results in Altered Signaling Characteristics in the DA

**Figure 6.**
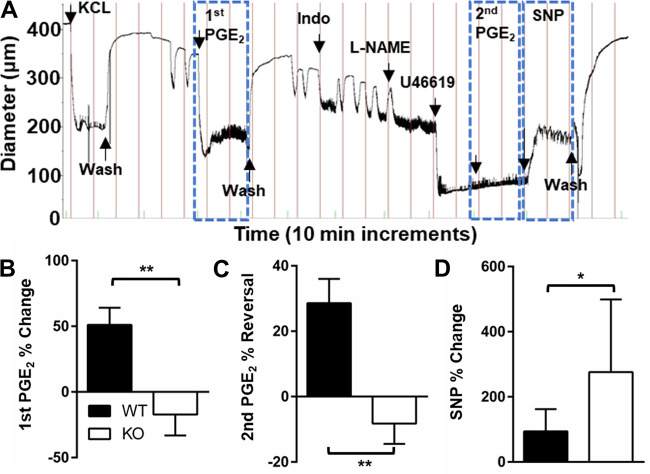
Patent ductus arteriosus (PDA) of prostaglandin E_2_ (PGE_2_) receptor EP_4_ knockout (EP_4_ KO) mice exhibits an unexpected contractile response to PGE_2_. *A*: representative tracing of an isolated EP_4_ KO ductus arteriosus (DA) exposed to various stimuli demonstrating unexpected PGE-induced vasoconstriction (10^−6^ M), but appropriate contractile response to the thromboxane mimetic U46619 (10^−5^ M) and vasodilatory response to the nitric oxide (NO) donor sodium nitroprusside (SNP; 10^−5^ M). *B* and *C*: cumulative results in wild-type (WT) littermate DAs demonstrate the typical PGE_2_-mediated vasodilatory response in comparison with PGE_2_-induced constriction in EP_4_ KO DAs under baseline (*B*) and U46619-preconstricted (*C*) conditions, done in the presence of 10^−5^ M indomethacin and 10^−4^ M *N*^G^-nitro-l-arginine methyl ester (l-NAME) to inhibit endogenous PGE_2_ and NO production. *D*: subsequent exposure of the preconstricted and PGE_2_- and NO-inhibited DA to SNP resulted in greater DA dilation in EP_4_ KO than WT DAs (WT, *n* = 13; KO, *n* = 13, *B–D*). *P < 0.05 and **P < 0.01 compared with WT (*B–D*, *t* test).

To assess the functional consequences of EP_4_ deletion in determining key aspects of DA signaling, we used pressurized vessel myography. Isolated EP_4_ KO (B6.129S6-*Ptger4*^tm1.2Matb^) DAs were mounted and their response to different vasoactive stimuli were interrogated (Fig. 6*A*). Unexpectedly, EP_4_ KO DAs showed significant constriction in response to PGE_2_ exposure in both nonpreconstricted and preconstricted states when endogenous PG and NO synthesis were suppressed (Fig. 6, *B* and *C*). These findings stand in contrast to the strong dilation normally induced by PGE_2_ in the DA of most species. The unanticipated contractile response of EP_4_ KO DAs to PGE_2_ may be related to reduction in the vasodilatory EP_2_ receptor combined with the loss of EP_4_, and preservation of EP_1_ and EP_3_ receptor expression, which typically mediate vasoconstrictive responses (Supplemental Fig. S4). This might also be explained by shifting receptor affinity or receptor density among the remaining EP receptors, as EP_4_ is known to drive a positive feedback loop of PGE_2_ production (65). Furthermore, the change in downstream signaling resulting from the absence of EP_4_ may affect the contractile apparatus of the DA but was not addressed in the present studies. EP_4_ KO (B6.129S6-Ptger4^tm1.2Matb^) DAs also showed an enhanced vasodilatory response to the NO donor sodium nitroprusside (SNP), suggesting that altered vascular effects are not limited to the prostanoid pathway (Fig. 6*D*) and may have NO-dominant regulation similar to the preterm DA (Fig. 3, *C* and *D*).

### Deletion of EP_4_ Results in Altered DA Vasoconstrictive Properties

**Figure 7.**
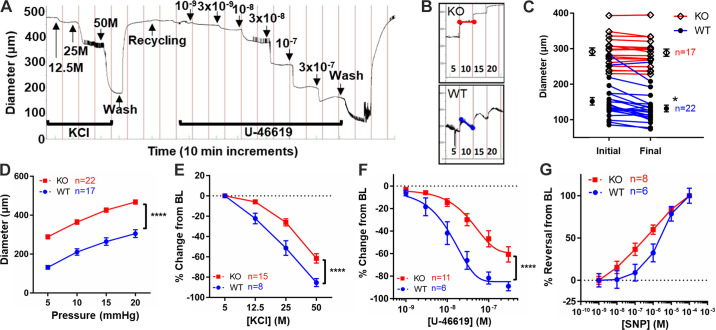
Patent ductus arteriosus (PDA) of prostaglandin E_2_ (PGE_2_) receptor EP_4_ knockout (EP_4_ KO) mice exhibits impaired responses to multiple stimuli. *A*: representative tracing of an isolated EP_4_ KO DA in response to U46619 contractile stimuli. *B*: representative tracings of pressure-induced tone (myogenic response) during initial pressure ramps in EP_4_ KO and wild-type (WT) littermate vessels. *C*: plot showing impaired myogenic response in EP_4_ KO ductus arteriosus (DAs) (10–15 mm step). *D–G*: when compared with WT littermates, the ex vivo EP_4_ KO DA was consistently noted to have larger diameter at each pressure step (*D*), reduced contractile response to membrane depolarization under high KCl conditions (*E*), reduced contraction in response to the thromboxane receptor agonist U46619 (*F*), and enhanced vasodilatory response to sodium nitroprusside (SNP; *G*). BL, baseline. *P < 0.05 and ****P < 0.0001. (*C*, ANOVA; *D–G*, two-way ANOVA).

To further interrogate the impact of EP_4_ loss, we examined dynamic responses of the isolated DA in a second EP_4_ KO model (B6;129-*Ptger4*^tm1Sna^) (Fig. 7*A*). The DA of WT offspring demonstrated consistent pressure-induced tone (myogenic response) during pressure ramps at the start of each study, whereas littermate EP_4_ KO (B6;129-*Ptger4*^tm1Sna^) DAs showed little or no myogenic response (Fig. 7, *B* and *C*). EP_4_ KO (B6;129-*Ptger4*^tm1Sna^) DAs had significantly greater lumen diameter, nearly twice that of WT littermates, at each pressure step (Fig. 7*D*). Both of these findings are consistent with a decrease in basal DA tone. We did not observe that the DA of EP_4_ KO mice was physically larger, as the DA was appropriately proportioned to the Ao and pulmonary artery during dissection (Supplemental Fig. S4*A*), and EP_4_ KO pups were no larger than WT littermates (Supplemental Fig. S4*B*). To test whether this reduction in tone corresponded with decreased vasconstrictive potential, EP_4_ KO (B6;129-*Ptger4*^tm1Sna^) DAs were exposed to different vasoactive stimuli. EP_4_ KO DAs exhibited decreases in KCl- and U46619-induced vasoconstriction compared with WT littermates, suggesting the loss of EP_4_ adversely impacts voltage-gated and agonist-induced vasoconstrictive pathways in the DA (Fig. 7, *E* and *F*). Interestingly, the EP_4_ KO DA also showed an increased trend in responsiveness to SNP, consistent with a continued importance for NO as a vasodilator in the KO DA (Fig. 7*G*), similar to premature WT DAs (Fig. 3, *C* and *D*).

### Deletion of EP_4_ Does Not Impair DA Response to O_2_ but Reveals a Premature Phenotype

**Figure 8.**
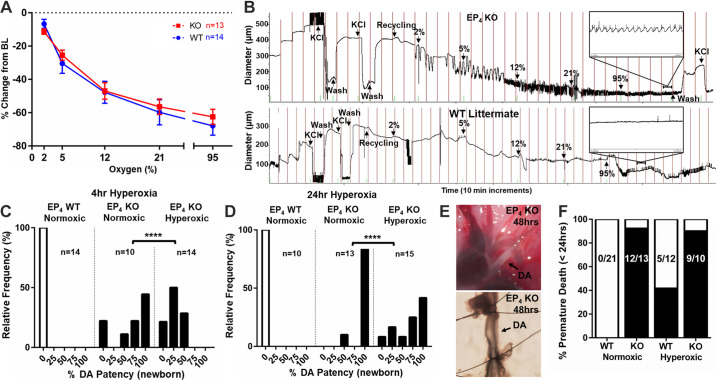
Patent ductus arteriosus (PDA) of prostaglandin E_2_ (PGE_2_) receptor EP_4_-knockout (EP_4_ KO) mice responds effectively to O_2_ but displays an immature phenotype. *A*: no difference was noted between ex vivo wild-type (WT) littermate and EP_4_ KO ductus arteriosus (DAs) after exposure to increasing O_2_ concentrations. BL, baseline. *B*: despite their similar degrees of constriction, EP_4_ KO DAs were consistently observed to have O_2_-induced vasomotion (*B*, *inset*), a characteristic of premature DAs which was not observed in WT littermate DAs (*B*, *bottom*). *C* and *D*: in vivo studies to test O_2_-induced DA constriction showed that short-term (4 h; *C*) and long-term (24 h; *D*) exposure to hyperoxia lead to significant constriction of the EP_4_ KO DA. *E*: surviving EP_4_ KO offspring exposed to hyperoxia for 48 h had constricted DAs that failed to remodel (*top*) and were easily reopened with minimal pressure once mounted in myography chambers (*bottom*). *F*: hyperoxia failed to increase survival in EP_4_ KO pups (*n* values superimposed on columns). ****P < 0.001. (*A*, two-way ANOVA; *C* and *D*, χ^2^).

Pressurized vessel myography was used to assess the ex vivo response of the EP_4_ KO DA to O_2_. Contrary to expectation, EP_4_ KO (B6;129-*Ptger4*^tm1Sna^) DAs exhibited no deficits in the O_2_-induced DA contractile response compared with WT (Fig. 8, *A* and *B*). Of interest, both KO models exhibited highly regular patterns of rhythmic constrictions and dilations with a peak–peak length of ∼10 s (Fig. 8*B*). This activity is consistent with vasomotion, a spontaneous oscillation in vessel tone or diameter noted in various vascular beds, which has only been previously described in the DA of premature (*D15*) mice (66, 67) .

To determine whether the seemingly intact O_2_ response of the ex vivo KO DA could effectively constrict the DA in vivo, we tested the effects of hyperoxia on DA closure and survival. Rearing EP_4_ KO (B6;129-*Ptger4*^tm1Sna^) pups in a hyperoxic chamber (∼70% O_2_) for 4 h lead to significant constriction of the EP_4_ KO DA (Fig. 8*C*). Some EP_4_ KO pups were then fostered to CD1 WT mothers in hyperoxia chambers (∼70% O_2_) for 24 or 48 h. Despite a significant constriction of the KO DA at 24 h (Fig. 8*D*), pup survival was not significantly increased (Fig. 8*F*). Incompletely constricted DAs were noted among the few surviving KO pups at 48 h, but their small DA lumens dilated when mounted for myography, consistent with failed permanent remodeling despite muscular constriction after extended O_2_ exposure (Fig. 8*E*). Together, these data suggest that impaired O_2_-induced DA constriction is likely not the cause of PDA in these neonates, and that hyperoxia can only partially rescue this phenotype.

The isolated DA of WT preterm fetal mice typically has a blunted ex vivo response to increasing O_2_ tension compared with term (43, 67). Interestingly, EP_4_ KO (B6;129-*Ptger4*^tm1Sna^) DAs displayed intact O_2_-induced DA constriction similar to term vessels, but also consistently demonstrated contraction-associated vasomotion, a reliable characteristic of isolated preterm mouse DAs. Despite an incomplete understanding of DA maturation and a paucity of developmental markers, we examined a select group of genes with known gestation-specific changes in the DA (13, 68, 69). Similar to the mixed O_2_ response phenotype, EP_4_ KO (B6;129-*Ptger4*^tm1Sna^) DAs shared a mature gene expression profile for some DA regulatory genes (*Tfap2b*, *IL15*, *Kcnma1*, *Kcnmb1*) but an immature profile for other DA-associated genes (*Pcp4*, *Rgs5*, *Des*) compared with WT littermates (Supplemental Fig. S5).

## DISCUSSION

In this study, we establish the acute role of EP_4_ as the primary EP receptor in the DA and primary regulator of DA tone in late gestation. We also provide new evidence for the chronic role of EP_4_ as a critical regulator of DA development and maturity.

Previous studies have determined that EP_4_ is the primary EP receptor in the DA of rats (10), rabbits (70), lambs (71, 72), pigs (73, 74), baboons (72), and humans (11, 12) and that EP_4_ is differentially expressed between DA and Ao in both mice (13), rodents, and humans (14). We found that EP_4_ expression rose over gestation and was limited to the media and intima of the DA, reflecting the human expression pattern (12). Decreasing expression of EP_4_ in the neonatal period, which we observed in both C57BL/6J and CD1 mice, has also been reported (70). The acute role of PGE as a dilator of the DA is also well established in rodents (28, 75–77) and used therapeutically for neonates born with ductus-dependent congenital cardiac defects (78–82). That said, the importance of PGE_2_ for DA tone seems to change throughout gestation. It was previously shown that PGE_2_ is more critical for DA patency in late gestation as opposed to midgestation, whereas NO is more critical in midgestation (43, 51, 83, 84). Our results show a gradual shift in these signals’ role in tone in the mouse DA, with a discernable plateau on *D17*.

Although the acute role of PGE_2_-EP_4_ in mediating DA tone is fairly straightforward, the chronic role of these signals in the DA has been a source of confusion and speculation. Multiple reports suggest that indomethacin tocolysis is associated with postnatal PDA (22, 85–93), although this has not been a consistent finding (94–96). This discrepancy is likely due to variations in the timing, dosage, and length of tocolytic treatments. In general, tocolytic treatments later in pregnancy (22, 87), closer to delivery (90), with a higher dosage (92), or longer duration (91, 92) promote PDA. Incidence aside, tocolysis has also been associated with failed pharmacological treatment and an increased need for ligation (22, 88, 89, 91, 93). These details offer insight into the role of PGE_2_ in DA development. The recapitulation of a PDA phenotype in mice with chronic COX inhibition solidified the importance of PGE_2_ in late- but not midgestation (24, 25). Despite the fact that KO models have been produced for all prostaglandin receptors (97–102), only EP_4_ KO mice have a PDA phenotype, and all global EP_4_ KO models have PDA (28–30). But it is counterintuitive that disruption of PGE_2_-EP_4_, a vasodilatory interaction in the DA, would result in a failure to constrict the DA of either tocolytic-exposed infants or KO mice. This discrepancy has been referred to as the “paradoxical PDA” (31) and suggests that EP_4_ has a distinct chronic role in DA development unrelated to the acute mediation of DA tone. Our group and others have proposed the existence of a developmental or transcriptional program that is necessary for establishing the mature, functional DA (103–107) with PGE_2_ considered as a potential regulator (24, 25). We found that antagonizing EP_4_ from *D17* to *D19* was sufficient to produce PDA. This corresponds with rising EP_4_ expression and a shift in dependence on PGE_2_-EP_4_ signaling for DA patency both on *D17*. This is likely the gestational point at which the PGE_2_-EP_4_-driven developmental program becomes active and begins to prepare the DA for constriction and remodeling after birth.

It remains unclear what PGE_2_-EP_4_ signaling does in the DA to prepare it for closure and remodeling. Microarray analysis of the EP_4_ KO DA found differential expression of genes associated with SMC phenotypes (32). These genes could be grouped into those affecting function of the contractile apparatus, migration, growth, and vascular tone. However, these changes in expression are likely linked to shifting proportions of particular phenotypes within the SMC population. Notably, α7 integrin, myocardin, and Myh11, genes associated with the contractile SMC phenotype (108), are downregulated in the KO (32). Similarly, matrix metallopeptidase genes, associated with the synthetic SMC phenotype (108), are upregulated in the KO (32). These data suggest that PGE_2_-EP_4_ contributes to establishing the phenotypic characteristics of the SMC population in the maturing DA required for both contraction and remodeling, although it should be noted there are many markers of SMC phenotype that did not significantly change in the KO DA (108–110) or in other transcriptional assessments of the murine DA (14, 69). This may partially explain our observation of decreased tone and decreased responsiveness of the EP_4_ KO DA to constrictive stimuli. SMC migration plays a key role in the permanent closure and fibromuscular remodeling of the DA (62–64, 111). During development, VSMCs migrate inward from the media, cross the increasingly fenestrated internal elastic lamina, and relocate into the subendothelial space where they form a neointima of radially realigned SMCs (36) in preparation for postnatal closure. In larger species, including humans, this includes the formation of large intimal cushions (61, 111) that facilitate occlusion of the constricting DA after birth. EP_4_ has been shown to promote PKA-independent SMC migration via EPAC1 (*Rapgef3*) in the rat DA (33), supporting our finding of a DA-specific effect of EP_4_ antagonism on SMC migration. Extensive studies demonstrate the role of EP_4_ stimulation in directing extracellular matrix composition in support of this process. EP_4_ stimulation has been shown to promote hyaluronic acid production leading to increased DA SMC migration (10). This is exacerbated by the inhibitor effects of EP_4_ stimulation on elastogenesis, supporting fenestration of the internal elastic lamina (35).

Given this context, it is a surprising but logical finding that hyperoxia-treated EP_4_ KO DAs were able to constrict but not remodel, in contrast to our original hypothesis. Recent studies have identified genes associated with remodeling of the mouse DA after birth that could be important for understanding remodeling deficits in the EP_4_ KO (112) but transcriptional analysis of postbirth KO DAs are not available for comparison. Interestingly, recent studies into the Prdm6-KO mouse model revealed both decreased EP_4_ expression and an inability to constrict in response to oxygen (113). At first, these results appear to contradict our findings, but previous studies of *D15* DAs found a partially intact oxygen response, indicating that the DA’s oxygen-sensing mechanism begins development before EP_4_ begins its developmental role. It is still unclear what factors regulate EP_4_ expression and timing in the DA, but Prdm6 is a known transcriptional repressor, and AP2 binding sites are present in the EP_4_ promoter, suggesting that Tfap2b may play a role.

Since the potential roles for EP_4_ in DA development are so diverse, we believe our data provide a novel conceptual framework to consider the KO phenotype in terms of DA maturity, with EP_4_ KO DAs more comparable with vessels from *D17* or earlier. Previous myography studies of premature mouse DAs have shown decreased responsiveness to KCl and PGE_2_, as well as the presence of vasomotion (66) all of which were present in EP_4_ KO DAs. In addition, the absence of effects of EP_4_ deletion on in utero patency could result from maintenance of NO-mediated dilation into late gestation. On the other hand, previous studies have indicated that immature DAs have a diminished O_2_ response (52), unlike the EP_4_ KO. This nuance coincides with our finding that three of seven maturity markers were more similar to premature vessels, whereas four were more similar to mature vessels. Thus, the EP_4_ KO DA appears to have an immature phenotype, but only with regard to distinct pathways. This immature phenotype is characterized in the KO DA by impairments in the first, muscular phase of DA closure, as well as previously described abnormalities in the second phase of permanent remodeling.

### Limitations

One limitation of this study, and the general use of mice to investigate PGE_2_-EP_4_ in the DA, is strain variation. Attempts to cross the EP_4_ KO allele onto a CD1 background resulted in a loss of the PDA phenotype. A similar loss of the EP_4_-associated PDA phenotype while outcrossing has been reported (37, 114), and the initial characterization of the EP_4_ KO on a mixed genetic background revealed an attenuated phenotype with successive generations of outcrossing (28). Similarly, attempts to induce a reliable PDA phenotype via pharmacological antagonism of the EP_4_ receptor were successful in C57BL/6J mice but were less effective in CD1 mice. In addition, WT littermates from our EP_4_ KO colony were no more susceptible to pharmacological antagonism of EP_4_ than C57BL/6J WTs, indicating that these are strain-specific effects, not a result of genetic drift within our colony. A relative decrease in EP receptor expression levels in CD1 compared with C57BL/6J DAs may be a possible explanation. Clearly, strain variation in EP expression was significant enough to alter susceptibility to PDA phenotypes. It is unclear what compensatory mechanisms maintain DA patency in utero and prepare the DA to function after birth. Our study was not designed to address the role that strain variation plays in the DA. Future studies using KO mice will need to be aware of the effects of background strain on PDA phenotype and outcomes.

Another limitation is the use of passaged primary SMCs to explore cell migration in the DA. Because of the size of the mouse DA, disaggregation of cells from tissue, or studies of unpassaged primary cells could not yield the quantity of cells necessary for our studies. Although the loss of phenotype is a common consequence of culturing primary cells with FBS or for multiple passages (115, 116), we tried to minimize these concerns by using low passage cells and serum starvation before experiments, similar to other DA SMC studies (33, 62–64, 117, 118). We found migratory differences between cultured DA and Ao cells, suggesting that some degree of tissue-specific identity was maintained. More thorough analyses of cultured DA SMCs in the future should use immortalized cells to minimize phenotypic drift and always include a biologically matched Ao control.

### Conclusion

In summary, our findings suggest that PGE_2_-EP_4_ signaling plays a crucial developmental role for the proper maturation and function of the term DA that is distinct from PGE’s widely known role in acute control of DA vasomotor tone. Similar to premature infants suffering PDA, EP_4_ KO mice are delivered without experiencing this late-gestational surge of EP_4_ expression and stimulation, which guides the expression of critical SMC genes, cell migration, and changes to ECM necessary for DA closure and remodeling at birth. As such, the EP_4_ KO DA is likely not a mature vessel with a single mechanistic defect, or a wholly immature vessel, but a partially matured vessel capable of weak transient constriction and incapable of permanently remodeling. Further exploration of EP_4_ function and DA development will likely hold key insights on the process of DA maturation relevant for human health.

## DATA AVAILABILITY

Data will be made available upon reasonable request.

## SUPPLEMENTAL DATA

10.6084/m9.figshare.23802282 Supplemental Figs. S1–S5 and Table S1: https://doi.org/10.6084/m9.figshare.23802282.

## GRANTS

This work was supported by National Institutes of Health (NIH) Grant HD007502 and American Heart Association (AHA) Predoctoral Fellowship Grant 18PRE34060243 (to M. T. Yarboro), NIH Grant HD099777 (to E. L. Shelton), and NIH Grants HL128386 and HL077395 and AHA Grant 0455360B (to J. Reese).

## DISCLOSURES

No conflicts of interest, financial or otherwise, are declared by the authors.

## AUTHOR CONTRIBUTIONS

M.T.Y., N.B., C.W.H., S.D.P., C.S.J., J.M.S.S., E.L.S., and J.R. conceived and designed research; M.T.Y., N.B., D.C.S., C.W.H., T.W., S.D.P., C.D.B., A.J.B., C.S.J., E.L.S., and J.R. performed experiments; M.T.Y., N.B., D.C.S., C.W.H., T.W., S.D.P., C.D.B., C.S.J., J.M.S.S., and J.R. analyzed data; M.T.Y., N.B., D.C.S., T.W., E.L.S., and J.R. interpreted results of experiments; M.T.Y., N.B., T.W., and J.R. prepared figures; M.T.Y. and J.R. drafted manuscript; M.T.Y., D.C.S., A.J.B., J.M.S.S., E.L.S., and J.R. edited and revised manuscript; M.T.Y., N.B., D.C.S., C.W.H., T.W., S.D.P., C.D.B., A.J.B., C.S.J., J.M.S.S., E.L.S., and J.R. approved final version of manuscript.

## References

[1] Crockett SL, Berger CD, Shelton EL, Reese J. Molecular and mechanical factors contributing to ductus arteriosus patency and closure. Congenit Heart Dis 14: 15–20, 2019. doi:10.1111/chd.12714. 30468303 PMC6393200

[2] Ovali F. Molecular and mechanical mechanisms regulating ductus arteriosus closure in preterm infants. Front Pediatr 8: 516, 2020.doi:10.3389/fped.2020.00516. 32984222 PMC7477801

[3] van der Linde D, Konings EE, Slager MA, Witsenburg M, Helbing WA, Takkenberg JJ, Roos-Hesselink JW. Birth prevalence of congenital heart disease worldwide: a systematic review and meta-analysis. J Am Coll Cardiol 58: 2241–2247, 2011. doi:10.1016/j.jacc.2011.08.025. 22078432

[4] Gillam-Krakauer M, Reese J. Diagnosis and management of patent ductus arteriosus. Neoreviews 19: e394–e402, 2018. doi:10.1542/neo.19-7-e394. 30505242 PMC6269146

[5] Hammerman C. Patent ductus arteriosus. Clinical relevance of prostaglandins and prostaglandin inhibitors in PDA pathophysiology and treatment. Clin Perinatol 22: 457–479, 1995. 7671547

[6] Vane SJ. Differential inhibition of cyclooxygenase isoforms: an explanation of the action of NSAIDs. J Clin Rheumatol 4: s3–s10, 1998. doi:10.1097/00124743-199810001-00002. 19078319

[7] Sugimoto Y, Narumiya S. Prostaglandin E receptors. J Biol Chem 282: 11613–11617, 2007. doi:10.1074/jbc.R600038200. 17329241

[8] Konya V, Marsche G, Schuligoi R, Heinemann A. E-type prostanoid receptor 4 (EP4) in disease and therapy. Pharmacol Ther 138: 485–502, 2013. doi:10.1016/j.pharmthera.2013.03.006. 23523686 PMC3661976

[9] Yokoyama U, Iwatsubo K, Umemura M, Fujita T, Ishikawa Y. The prostanoid EP4 receptor and its signaling pathway. Pharmacol Rev 65: 1010–1052, 2013. doi:10.1124/pr.112.007195. 23776144

[10] Yokoyama U, Minamisawa S, Quan H, Ghatak S, Akaike T, Segi-Nishida E, Iwasaki S, Iwamoto M, Misra S, Tamura K, Hori H, Yokota S, Toole BP, Sugimoto Y, Ishikawa Y. Chronic activation of the prostaglandin receptor EP4 promotes hyaluronan-mediated neointimal formation in the ductus arteriosus. J Clin Invest 116: 3026–3034, 2006. doi:10.1172/JCI28639. 17080198 PMC1626128

[11] Leonhardt A, Glaser A, Wegmann M, Schranz D, Seyberth H, Nüsing R. Expression of prostanoid receptors in human ductus arteriosus. Br J Pharmacol 138: 655–659, 2003. doi:10.1038/sj.bjp.0705092. 12598419 PMC1573700

[12] Rheinlaender C, Weber SC, Sarioglu N, Strauss E, Obladen M, Koehne P. Changing expression of cyclooxygenases and prostaglandin receptor EP4 during development of the human ductus arteriosus. Pediatr Res 60: 270–275, 2006. doi:10.1203/01.pdr.0000233066.28496.7c. 16857763

[13] Shelton EL, Ector G, Galindo CL, Hooper CW, Brown N, Wilkerson I, Pfaltzgraff ER, Paria BC, Cotton RB, Stoller JZ, Reese J. Transcriptional profiling reveals ductus arteriosus-specific genes that regulate vascular tone. Physiol Genomics 46: 457–466, 2014. doi:10.1152/physiolgenomics.00171.2013. 24790087 PMC4080279

[14] Yarboro MT, Durbin MD, Herington JL, Shelton EL, Zhang T, Ebby CG, Stoller JZ, Clyman RI, Reese J. Transcriptional profiling of the ductus arteriosus: comparison of rodent microarrays and human RNA sequencing. Semin Perinatol 42: 212–220, 2018. doi:10.1053/j.semperi.2018.05.003. 29910032 PMC6064668

[15] Clyman RI, Chan CY, Mauray F, Chen YQ, Cox W, Seidner SR, Lord EM, Weiss H, Waleh N, Evans SM, Koch CJ. Permanent anatomic closure of the ductus arteriosus in newborn baboons: the roles of postnatal constriction, hypoxia, and gestation. Pediatr Res 45: 19–29, 1999. doi:10.1203/00006450-199901000-00005. 9890604

[16] Stoller JZ, Demauro SB, Dagle JM, Reese J. Current perspectives on pathobiology of the ductus arteriosus. J Clin Exp Cardiolog 8: S8-001, 2012. doi:10.4172/2155-9880.S8-001. 23519783 PMC3601484

[17] Bentley RET, Hindmarch CCT, Dunham-Snary KJ, Snetsinger B, Mewburn JD, Thébaud A, Lima PDA, Thébaud B, Archer SL. The comprehensive transcriptome of human ductus arteriosus smooth muscle cells (hDASMC). Data Brief 40: 107736, 2022. doi:10.1016/j.dib.2021.107736. 35005134 PMC8717140

[18] Coggins KG, Latour A, Nguyen MS, Audoly L, Coffman TM, Koller BH. Metabolism of PGE2 by prostaglandin dehydrogenase is essential for remodeling the ductus arteriosus. Nat Med 8: 91–92, 2002. doi:10.1038/nm0202-91. 11821873

[19] Roizen JD, Asada M, Tong M, Tai HH, Muglia LJ. Preterm birth without progesterone withdrawal in 15-hydroxyprostaglandin dehydrogenase hypomorphic mice. Mol Endocrinol 22: 105–112, 2008. doi:10.1210/me.2007-0178. 17872381 PMC2194629

[20] Moise KJ Jr. Effect of advancing gestational age on the frequency of fetal ductal constriction in association with maternal indomethacin use. Am J Obstet Gynecol 168: 1350–1353, 1993. doi:10.1016/s0002-9378(11)90763-7. 8498410

[21] Vermillion ST, Scardo JA, Lashus AG, Wiles HB. The effect of indomethacin tocolysis on fetal ductus arteriosus constriction with advancing gestational age. Am J Obstet Gynecol 177: 256–259; discussion 259–261, 1997. doi:10.1016/s0002-9378(97)70184-4. 9290437

[22] Norton ME, Merrill J, Cooper BA, Kuller JA, Clyman RI. Neonatal complications after the administration of indomethacin for preterm labor. N Engl J Med 329: 1602–1607, 1993. doi:10.1056/NEJM199311253292202. 8232428

[23] Loftin CD, Trivedi DB, Langenbach R. Cyclooxygenase-1-selective inhibition prolongs gestation in mice without adverse effects on the ductus arteriosus. J Clin Invest 110: 549–557, 2002. doi:10.1172/JCI14924. 12189249 PMC150416

[24] Reese J, Anderson JD, Brown N, Roman C, Clyman RI. Inhibition of cyclooxygenase isoforms in late- but not midgestation decreases contractility of the ductus arteriosus and prevents postnatal closure in mice. Am J Physiol Regul Integr Comp Physiol 291: R1717–R1723, 2006. doi:10.1152/ajpregu.00259.2006. 16857891 PMC2819844

[25] Reese J, Waleh N, Poole SD, Brown N, Roman C, Clyman RI. Chronic in utero cyclooxygenase inhibition alters PGE2-regulated ductus arteriosus contractile pathways and prevents postnatal closure. Pediatr Res 66: 155–161, 2009. doi:10.1203/PDR.0b013e3181aa07eb. 19390487 PMC3066019

[26] Reese J, Paria BC, Brown N, Zhao X, Morrow JD, Dey SK. Coordinated regulation of fetal and maternal prostaglandins directs successful birth and postnatal adaptation in the mouse. Proc Natl Acad Sci USA 97: 9759–9764, 2000. doi:10.1073/pnas.97.17.9759. 10944235 PMC16938

[27] Loftin CD, Trivedi DB, Tiano HF, Clark JA, Lee CA, Epstein JA, Morham SG, Breyer MD, Nguyen M, Hawkins BM, Goulet JL, Smithies O, Koller BH, Langenbach R. Failure of ductus arteriosus closure and remodeling in neonatal mice deficient in cyclooxygenase-1 and cyclooxygenase-2. Proc Natl Acad Sci USA 98: 1059–1064, 2001. doi:10.1073/pnas.98.3.1059. 11158594 PMC14708

[28] Nguyen M, Camenisch T, Snouwaert JN, Hicks E, Coffman TM, Anderson PA, Malouf NN, Koller BH. The prostaglandin receptor EP4 triggers remodelling of the cardiovascular system at birth. Nature 390: 78–81, 1997. doi:10.1038/36342. 9363893

[29] Segi E, Sugimoto Y, Yamasaki A, Aze Y, Oida H, Nishimura T, Murata T, Matsuoka T, Ushikubi F, Hirose M, Tanaka T, Yoshida N, Narumiya S, Ichikawa A. Patent ductus arteriosus and neonatal death in prostaglandin receptor EP4-deficient mice. Biochem Biophys Res Commun 246: 7–12, 1998. doi:10.1006/bbrc.1998.8461. 9600059

[30] Schneider A, Guan Y, Zhang Y, Magnuson MA, Pettepher C, Loftin CD, Langenbach R, Breyer RM, Breyer MD. Generation of a conditional allele of the mouse prostaglandin EP4 receptor. Genesis 40: 7–14, 2004. doi:10.1002/gene.20048. 15354288

[31] Ivey KN, Srivastava D. The paradoxical patent ductus arteriosus. J Clin Invest 116: 2863–2865, 2006. doi:10.1172/JCI30349. 17080192 PMC1626120

[32] Gruzdev A, Nguyen M, Kovarova M, Koller BH. PGE2 through the EP4 receptor controls smooth muscle gene expression patterns in the ductus arteriosus critical for remodeling at birth. Prostaglandins Other Lipid Mediat 97: 109–119, 2012. doi:10.1016/j.prostaglandins.2012.02.001. 22342504 PMC3312001

[33] Yokoyama U, Minamisawa S, Quan H, Akaike T, Suzuki S, Jin M, Jiao Q, Watanabe M, Otsu K, Iwasaki S, Nishimaki S, Sato M, Ishikawa Y. Prostaglandin E2-activated Epac promotes neointimal formation of the rat ductus arteriosus by a process distinct from that of cAMP-dependent protein kinase A. J Biol Chem 283: 28702–28709, 2008. doi:10.1074/jbc.M804223200. 18697745 PMC2568928

[34] Yokoyama U, Minamisawa S, Katayama A, Tang T, Suzuki S, Iwatsubo K, Iwasaki S, Kurotani R, Okumura S, Sato M, Yokota S, Hammond HK, Ishikawa Y. Differential regulation of vascular tone and remodeling via stimulation of type 2 and type 6 adenylyl cyclases in the ductus arteriosus. Circ Res 106: 1882–1892, 2010. doi:10.1161/CIRCRESAHA.109.214924. 20431059 PMC2892563

[35] Yokoyama U, Minamisawa S, Shioda A, Ishiwata R, Jin MH, Masuda M, Asou T, Sugimoto Y, Aoki H, Nakamura T, Ishikawa Y. Prostaglandin E2 inhibits elastogenesis in the ductus arteriosus via EP4 signaling. Circulation 129: 487–496, 2014. doi:10.1161/CIRCULATIONAHA.113.004726. 24146253

[36] Yokoyama U. Prostaglandin E-mediated molecular mechanisms driving remodeling of the ductus arteriosus. Pediatr Int 57: 820–827, 2015. doi:10.1111/ped.12769. 26228894

[37] Kraemer MP, Choi H, Reese J, Lamb FS, Breyer RM. Regulation of arterial reactivity by concurrent signaling through the E-prostanoid receptor 3 and angiotensin receptor 1. Vascul Pharmacol 84: 47–54, 2016. doi:10.1016/j.vph.2016.05.015. 27260940 PMC4976016

[38] Flinsenberg TW, van der Sterren S, van Cleef AN, Schuurman MJ, Agren P, Villamor E. Effects of sex and estrogen on chicken ductus arteriosus reactivity. Am J Physiol Regul Integr Comp Physiol 298: R1217–R1224, 2010. doi:10.1152/ajpregu.00839.2009. 20164203

[39] Boatwright NH, Sorenson C, Israel S, Yarboro MT, Su R, Tolentino CD, Paria BC, Clark RH, Shelton EL, Reese J. Limited contribution of sex and sex hormones to regulation of the mouse ductus arteriosus (DA) and human PDA. Experimental Biology (EPAS) Conference, Denver, CO, Apr 21–25, 2022, Abstract Number 211.220.

[40] Borges-Lujan M, Gonzalez-Luis GE, Roosen T, Huizing MJ, Villamor E. Sex differences in patent ductus arteriosus incidence and response to pharmacological treatment in preterm infants: a systematic review, meta-analysis and meta-regression. J Pers Med 12: 1143, 2022. doi:10.3390/jpm12071143. 35887640 PMC9321725

[41] Villamor E, Borges-Luján M, González-Luis G. Association of patent ductus arteriosus with fetal factors and endotypes of prematurity. Semin Perinatol 47: 151717, 2023. doi:10.1016/j.semperi.2023.151717. 36914506

[42] Crockett SL, Harris M, Boatwright N, Su RL, Yarboro MT, Berger CD, Shelton EL, Reese J, Segar JL. Role of dopamine and selective dopamine receptor agonists on mouse ductus arteriosus tone and responsiveness. Pediatr Res 87: 991–997, 2020. doi:10.1038/s41390-019-0716-x. 31816622 PMC7196482

[43] Reese J, O’Mara PW, Poole SD, Brown N, Tolentino C, Eckman DM, Aschner JL. Regulation of the fetal mouse ductus arteriosus is dependent on interaction of nitric oxide and COX enzymes in the ductal wall. Prostaglandins Other Lipid Mediat 88: 89–96, 2009. doi:10.1016/j.prostaglandins.2008.11.001. 19049898 PMC2813040

[44] Guan Y, Zhang Y, Wu J, Qi Z, Yang G, Dou D, Gao Y, Chen L, Zhang X, Davis LS, Wei M, Fan X, Carmosino M, Hao C, Imig JD, Breyer RM, Breyer MD. Antihypertensive effects of selective prostaglandin E2 receptor subtype 1 targeting. J Clin Invest 117: 2496–2505, 2007. doi:10.1172/JCI29838. 17710229 PMC1940235

[45] Sun Y, Zhang Y, Zhu Y, Zhang A, Huang S, Yin X, Ding G, Liu M, Jia Z. Inhibition of mitochondrial complex-1 restores the downregulation of aquaporins in obstructive nephropathy. Am J Physiol Renal Physiol 311: F777–F786, 2016. doi:10.1152/ajprenal.00215.2015. 27413198

[46] Ma X, Aoki T, Tsuruyama T, Narumiya S. Definition of prostaglandin E2-EP2 signals in the colon tumor microenvironment that amplify inflammation and tumor growth. Cancer Res 75: 2822–2832, 2015. doi:10.1158/0008-5472.CAN-15-0125. 26018088

[47] Li C, Liu X, Liu Y, Zhang E, Medepalli K, Masuda K, Li N, Wikenheiser-Brokamp KA, Osterburg A, Borchers MT, Kopras EJ, Plas DR, Sun J, Franz DN, Capal JK, Mays M, Sun Y, Kwiatkowski DJ, Alayev A, Holz MK, Krueger DA, Siroky BJ, Yu JJ. Tuberin regulates prostaglandin receptor-mediated viability, via Rheb, in mTORC1-hyperactive cells. Mol Cancer Res 15: 1318–1330, 2017. doi:10.1158/1541-7786.MCR-17-0077. 28710231

[48] Machwate M, Harada S, Leu CT, Seedor G, Labelle M, Gallant M, Hutchins S, Lachance N, Sawyer N, Slipetz D, Metters KM, Rodan SB, Young R, Rodan GA. Prostaglandin receptor EP_4_ mediates the bone anabolic effects of PGE_2_. Mol Pharmacol 60: 36–41, 2001 [Erratum in *Mol Pharmacol* 64: 192, 2003]. doi:10.1124/mol.60.1.36. 11408598

[49] Xu S, Zhang Z, Ogawa O, Yoshikawa T, Sakamoto H, Shibasaki N, Goto T, Wang L, Terada N. An EP4 antagonist ONO-AE3-208 suppresses cell invasion, migration, and metastasis of prostate cancer. Cell Biochem Biophys 70: 521–527, 2014. doi:10.1007/s12013-014-9951-2. 24744183

[50] Momma K, Toyoshima K, Takeuchi D, Imamura S, Nakanishi T. In vivo constriction of the fetal and neonatal ductus arteriosus by a prostanoid EP4-receptor antagonist in rats. Pediatr Res 58: 971–975, 2005. doi:10.1203/01.pdr.0000182182.49476.24. 16257930

[51] Richard C, Gao J, LaFleur B, Christman BW, Anderson J, Brown N, Reese J. Patency of the preterm fetal ductus arteriosus is regulated by endothelial nitric oxide synthase and is independent of vasa vasorum in the mouse. Am J Physiol Regul Integr Comp Physiol 287: R652–R660, 2004. doi:10.1152/ajpregu.00049.2004. 15142832

[52] Vucovich MM, Cotton RB, Shelton EL, Goettel JA, Ehinger NJ, Poole SD, Brown N, Wynn JL, Paria BC, Slaughter JC, Clark RH, Rojas MA, Reese J. Aminoglycoside-mediated relaxation of the ductus arteriosus in sepsis-associated PDA. Am J Physiol Heart Circ Physiol 307: H732–H740, 2014. doi:10.1152/ajpheart.00838.2013. 24993047 PMC4187398

[53] Hooper CW, Delaney C, Streeter T, Yarboro MT, Poole S, Brown N, Slaughter JC, Cotton RB, Reese J, Shelton EL. Selective serotonin reuptake inhibitor exposure constricts the mouse ductus arteriosus in utero. Am J Physiol Heart Circ Physiol 311: H572–H581, 2016. doi:10.1152/ajpheart.00822.2015. 27371685 PMC5142184

[54] Toyoshima K, Takeda A, Imamura S, Nakanishi T, Momma K. Constriction of the ductus arteriosus by selective inhibition of cyclooxygenase-1 and -2 in near-term and preterm fetal rats. Prostaglandins Other Lipid Mediat 79: 34–42, 2006. doi:10.1016/j.prostaglandins.2004.11.005. 16516808

[55] Coceani F, Liu Y, Seidlitz E, Kelsey L, Kuwaki T, Ackerley C, Yanagisawa M. Endothelin A receptor is necessary for O_2_ constriction but not closure of ductus arteriosus. Am J Physiol Heart Circ Physiol 277: H1521–H1531, 1999. doi:10.1152/ajpheart.1999.277.4.H1521. 10516191

[56] Katsuda S, Okada Y, Nakanishi I, Tanaka J. Inhibitory effect of dimethyl sulfoxide on the proliferation of cultured arterial smooth muscle cells: relationship to the cytoplasmic microtubules. Exp Mol Pathol 48: 48–58, 1988. doi:10.1016/0014-4800(88)90045-7. 3335251

[57] Galvao J, Davis B, Tilley M, Normando E, Duchen MR, Cordeiro MF. Unexpected low-dose toxicity of the universal solvent DMSO. FASEB J 28: 1317–1330, 2014. doi:10.1096/fj.13-235440. 24327606

[58] Rao R, Redha R, Macias-Perez I, Su Y, Hao C, Zent R, Breyer MD, Pozzi A. Prostaglandin E2-EP4 receptor promotes endothelial cell migration via ERK activation and angiogenesis in vivo. J Biol Chem 282: 16959–16968, 2007. doi:10.1074/jbc.M701214200. 17401137

[59] Ke J, Yang Y, Che Q, Jiang F, Wang H, Chen Z, Zhu M, Tong H, Zhang H, Yan X, Wang X, Wang F, Liu Y, Dai C, Wan X. Prostaglandin E2 (PGE2) promotes proliferation and invasion by enhancing SUMO-1 activity via EP4 receptor in endometrial cancer. Tumour Biol 37: 12203–12211, 2016. doi:10.1007/s13277-016-5087-x. 27230680 PMC5080328

[60] Osawa K, Umemura M, Nakakaji R, Tanaka R, Islam RM, Nagasako A, Fujita T, Yokoyama U, Koizumi T, Mitsudo K, Ishikawa Y. Prostaglandin E_2_ receptor EP4 regulates cell migration through Orai1. Cancer Sci 111: 160–174, 2020. doi:10.1111/cas.14247. 31755615 PMC6942437

[61] Tada T, Kishimoto H. Ultrastructural and histological studies on closure of the mouse ductus arteriosus. Acta Anat (Basel) 139: 326–334, 1990. doi:10.1159/000147020. 2075800

[62] Boudreau N, Turley E, Rabinovitch M. Fibronectin, hyaluronan, and a hyaluronan binding protein contribute to increased ductus arteriosus smooth muscle cell migration. Dev Biol 143: 235–247, 1991. doi:10.1016/0012-1606(91)90074-d. 1703972

[63] Clyman RI, Mauray F, Kramer RH. *β*_1_ and *β*_3_ integrins have different roles in the adhesion and migration of vascular smooth muscle cells on extracellular matrix. Exp Cell Res 200: 272–284, 1992. doi:10.1016/0014-4827(92)90173-6. 1374036

[64] Koppel R, Rabinovitch M. Regulation of fetal lamb ductus arteriosus smooth muscle cell migration by indomethacin and dexamethasone. Pediatr Res 33: 352–358, 1993. doi:10.1203/00006450-199304000-00009. 8479815

[65] Faour WH, Gomi K, Kennedy CR. PGE_2_ induces COX-2 expression in podocytes via the EP_4_ receptor through a PKA-independent mechanism. Cell Signal 20: 2156–2164, 2008. doi:10.1016/j.cellsig.2008.08.007. 18762248

[66] Vucovich M, Ehinger N, Poole SD, Lamb FS, Reese J. Spontaneous rhythmic contractions (vasomotion) of the isolated, pressurized ductus arteriosus of preterm, but not term, fetal mice. EJ Neonatol Res 2: 13–24, 2012. 23710420 PMC3661283

[67] Chen JX, O’Mara PW, Poole SD, Brown N, Ehinger NJ, Slaughter JC, Paria BC, Aschner JL, Reese J. Isoprostanes as physiological mediators of transition to newborn life: novel mechanisms regulating patency of the term and preterm ductus arteriosus. Pediatr Res 72: 122–128, 2012. doi:10.1038/pr.2012.58. 22565502 PMC3586272

[68] Jin MH, Yokoyama U, Sato Y, Shioda A, Jiao Q, Ishikawa Y, Minamisawa S. DNA microarray profiling identified a new role of growth hormone in vascular remodeling of rat ductus arteriosus. J Physiol Sci 61: 167–179, 2011. doi:10.1007/s12576-011-0133-3. 21287305 PMC10717642

[69] Yarboro MT, Gopal SH, Su RL, Morgan TM, Reese J. Mouse models of patent ductus arteriosus (PDA) and their relevance for human PDA. Dev Dyn 251: 424–443, 2022. doi:10.1002/dvdy.408. 34350653 PMC8814064

[70] Fan FL, Zhu S, Chen LH, Zou YL, Fan LH, Kang JH, Ma AQ, Guan YF. Role of prostaglandin E and its receptors in the process of ductus arteriosus maturation and functional closure in the rabbit. Clin Exp Pharmacol Physiol 37: 574–580, 2010. doi:10.1111/j.1440-1681.2010.05354.x. 20082631

[71] Smith GC, Wu WX, Nijland MJ, Koenen SV, Nathanielsz PW. Effect of gestational age, corticosteroids, and birth on expression of prostanoid EP receptor genes in lamb and baboon ductus arteriosus. J Cardiovasc Pharmacol 37: 697–704, 2001. doi:10.1097/00005344-200106000-00007. 11392466

[72] Waleh N, Kajino H, Marrache AM, Ginzinger D, Roman C, Seidner SR, Moss TJ, Fouron JC, Vazquez-Tello A, Chemtob S, Clyman RI. Prostaglandin E_2_—mediated relaxation of the ductus arteriosus: effects of gestational age on g protein-coupled receptor expression, signaling, and vasomotor control. Circulation 110: 2326–2332, 2004. doi:10.1161/01.CIR.0000145159.16637.5D. 15477420

[73] Bhattacharya M, Asselin P, Hardy P, Guerguerian AM, Shichi H, Hou X, Varma DR, Bouayad A, Fouron JC, Clyman RI, Chemtob S. Developmental changes in prostaglandin E_2_ receptor subtypes in porcine ductus arteriosus. Possible contribution in altered responsiveness to prostaglandin E_2_. Circulation 100: 1751–1756, 1999. doi:10.1161/01.cir.100.16.1751. 10525496

[74] Bouayad A, Bernier SG, Asselin P, Hardy P, Bhattacharya M, Quiniou C, Fouron JC, Guerguerian AM, Varma DR, Clyman RI, Chemtob S. Characterization of PGE2 receptors in fetal and newborn ductus arteriosus in the pig. Semin Perinatol 25: 70–75, 2001. doi:10.1053/sper.2001.23186. 11339668

[75] Sharpe GL, Thalme B, Larsson KS. Studies on closure of the ductus arteriosus. XI. Ductal closure in utero by a prostaglandin synthetase inhibitor. Prostaglandins 8: 363–368, 1974. doi:10.1016/0090-6980(74)90110-5. 4453621

[76] Sharpe GL, Larsson KS. Studies on closure of the ductus arteriosus. X. In vivo effect of prostaglandin. Prostaglandins 9: 703–719, 1975. doi:10.1016/0090-6980(75)90109-4. 240189

[77] Janatová T, Jarkovská D, Hruda J, Samánek M, Ostádal B. Effect of the administration of prostaglandins (PGE_2_) in the early postnatal period on closure of the ductus arteriosus in the laboratory rat. Physiol Bohemoslov 38: 201–206, 1989. 2528765

[78] Coceani F, Olley PM. The response of the ductus arteriosus to prostaglandins. Can J Physiol Pharmacol 51: 220–225, 1973. doi:10.1139/y73-031. 4705142

[79] Elliott RB, Starling MB, Neutze JM. Medical manipulation of the ductus arteriosus. Lancet 1: 140–142, 1975. doi:10.1016/s0140-6736(75)91432-4. 46053

[80] Olley PM, Coceani F, Bodach E. E-type prostaglandins: a new emergency therapy for certain cyanotic congenital heart malformations. Circulation 53: 728–731, 1976. doi:10.1161/01.cir.53.4.728. 56243

[81] Heymann MA, Rudolph AM. Ductus arteriosus dilatation by prostaglandin E_1_ in infants with pulmonary atresia. Pediatrics 59: 325–329, 1977. 840551

[82] Thanopoulos BD, Andreou A, Frimas C. Prostaglandin E2 administration in infants with ductus-dependent cyanotic congenital heart disease. Eur J Pediatr 146: 279–282, 1987. doi:10.1007/BF00716473. 3474149

[83] Clyman RI, Waleh N, Black SM, Riemer RK, Mauray F, Chen YQ. Regulation of ductus arteriosus patency by nitric oxide in fetal lambs: the role of gestation, oxygen tension, and vasa vasorum. Pediatr Res 43: 633–644, 1998. doi:10.1203/00006450-199805000-00012. 9585010

[84] Momma K, Toyono M. The role of nitric oxide in dilating the fetal ductus arteriosus in rats. Pediatr Res 46: 311–315, 1999. doi:10.1203/00006450-199909000-00010. 10473046

[85] Atad J, Lissak A, Rofe A, Abramovici H. Patent ductus arteriosus after prolonged treatment with indomethacin during pregnancy: case report. Int J Gynaecol Obstet 25: 73–76, 1987. doi:10.1016/0020-7292(87)90188-3. 2883050

[86] Moise KJ Jr, Huhta JC, Sharif DS, Ou CN, Kirshon B, Wasserstrum N, Cano L. Indomethacin in the treatment of premature labor. Effects on the fetal ductus arteriosus. N Engl J Med 319: 327–331, 1988. doi:10.1056/NEJM198808113190602. 3393194

[87] Gerson A, Abbasi S, Johnson A, Kalchbrenner M, Ashmead G, Bolognese R. Safety and efficacy of long-term tocolysis with indomethacin. Am J Perinatol 7: 71–74, 1990. doi:10.1055/s-2007-999450. 2294913

[88] Eronen M, Pesonen E, Kurki T, Teramo K, Ylikorkala O, Hallman M. Increased incidence of bronchopulmonary dysplasia after antenatal administration of indomethacin to prevent preterm labor. J Pediatr 124: 782–788, 1994. doi:10.1016/s0022-3476(05)81374-5. 8176569

[89] Hammerman C, Glaser J, Kaplan M, Schimmel MS, Ferber B, Eidelman AI. Indomethacin tocolysis increases postnatal patent ductus arteriosus severity. Pediatrics 102: E56, 1998. doi:10.1542/peds.102.5.e56. 9794986

[90] Souter D, Harding J, McCowan L, O'Donnell C, McLeay E, Baxendale H. Antenatal indomethacin–adverse fetal effects confirmed. Aust N Z J Obstet Gynaecol 38: 11–16, 1998. doi:10.1111/j.1479-828x.1998.tb02949.x. 9521382

[91] Suarez VR, Thompson LL, Jain V, Olson GL, Hankins GD, Belfort MA, Saade GR. The effect of in utero exposure to indomethacin on the need for surgical closure of a patent ductus arteriosus in the neonate. Am J Obstet Gynecol 187: 886–888, 2002. doi:10.1067/mob.2002.127464. 12388970

[92] Cordero L, Nankervis CA, Gardner D, Giannone PJ. The effects of indomethacin tocolysis on the postnatal response of the ductus arteriosus to indomethacin in extremely low birth weight infants. J Perinatol 27: 22–27, 2007. doi:10.1038/sj.jp.7211612. 17053778

[93] Soraisham AS, Dalgleish S, Singhal N. Antenatal indomethacin tocolysis is associated with an increased need for surgical ligation of patent ductus arteriosus in preterm infants. J Obstet Gynaecol Can 32: 435–442, 2010. doi:10.1016/S1701-2163(16)34496-6. 20500951

[94] Loe SM, Sanchez-Ramos L, Kaunitz AM. Assessing the neonatal safety of indomethacin tocolysis: a systematic review with meta-analysis. Obstet Gynecol 106: 173–179, 2005. doi:10.1097/01.AOG.0000168622.56478.df. 15994634

[95] Rovers JFJ, Thomissen IJC, Janssen LCE, Lingius S, Wieland BV, Dieleman JP, Niemarkt HJ, van Runnard Heimel PJ. The relationship between antenatal indomethacin as a tocolytic drug and neonatal outcomes: a retrospective cohort study. J Matern Fetal Neonatal Med 34: 2945–2951, 2021. doi:10.1080/14767058.2019.1674807. 31597542

[96] Turan OM, Driscoll C, Cetinkaya-Demir B, Gabbay-Benziv R, Turan S, Kopelman JN, Harman C. Prolonged early antenatal indomethacin exposure is safe for fetus and neonate. J Matern Fetal Neonatal Med 34: 167–176, 2021. doi:10.1080/14767058.2019.1599351. 30905227

[97] Sugimoto Y, Yamasaki A, Segi E, Tsuboi K, Aze Y, Nishimura T, Oida H, Yoshida N, Tanaka T, Katsuyama M, Hasumoto K, Murata T, Hirata M, Ushikubi F, Negishi M, Ichikawa A, Narumiya S. Failure of parturition in mice lacking the prostaglandin F receptor. Science 277: 681–683, 1997. doi:10.1126/science.277.5326.681. 9235889

[98] Murata T, Ushikubi F, Matsuoka T, Hirata M, Yamasaki A, Sugimoto Y, Ichikawa A, Aze Y, Tanaka T, Yoshida N, Ueno A, Oh-Ishi S, Narumiya S. Altered pain perception and inflammatory response in mice lacking prostacyclin receptor. Nature 388: 678–682, 1997. doi:10.1038/41780. 9262402

[99] Ushikubi F, Segi E, Sugimoto Y, Murata T, Matsuoka T, Kobayashi T, Hizaki H, Tuboi K, Katsuyama M, Ichikawa A, Tanaka T, Yoshida N, Narumiya S. Impaired febrile response in mice lacking the prostaglandin E receptor subtype EP3. Nature 395: 281–284, 1998. doi:10.1038/26233. 9751056

[100] Hizaki H, Segi E, Sugimoto Y, Hirose M, Saji T, Ushikubi F, Matsuoka T, Noda Y, Tanaka T, Yoshida N, Narumiya S, Ichikawa A. Abortive expansion of the cumulus and impaired fertility in mice lacking the prostaglandin E receptor subtype EP_2_. Proc Natl Acad Sci USA 96: 10501–10506, 1999. doi:10.1073/pnas.96.18.10501. 10468638 PMC17918

[101] Matsuoka T, Hirata M, Tanaka H, Takahashi Y, Murata T, Kabashima K, Sugimoto Y, Kobayashi T, Ushikubi F, Aze Y, Eguchi N, Urade Y, Yoshida N, Kimura K, Mizoguchi A, Honda Y, Nagai H, Narumiya S. Prostaglandin D2 as a mediator of allergic asthma. Science 287: 2013–2017, 2000. doi:10.1126/science.287.5460.2013. 10720327

[102] Okada Y, Hara A, Ma H, Xiao CY, Takahata O, Kohgo Y, Narumiya S, Ushikubi F. Characterization of prostanoid receptors mediating contraction of the gastric fundus and ileum: studies using mice deficient in prostanoid receptors. Br J Pharmacol 131: 745–755, 2000. doi:10.1038/sj.bjp.0703627. 11030724 PMC1572385

[103] Ivey KN, Sutcliffe D, Richardson J, Clyman RI, Garcia JA, Srivastava D. Transcriptional regulation during development of the ductus arteriosus. Circ Res 103: 388–395, 2008. doi:10.1161/CIRCRESAHA.108.180661. 18635823 PMC2645272

[104] Huang J, Cheng L, Li J, Chen M, Zhou D, Lu MM, Proweller A, Epstein JA, Parmacek MS. Myocardin regulates expression of contractile genes in smooth muscle cells and is required for closure of the ductus arteriosus in mice. J Clin Invest 118: 515–525, 2008. doi:10.1172/JCI33304. 18188448 PMC2176191

[105] Feng X, Krebs LT, Gridley T. Patent ductus arteriosus in mice with smooth muscle-specific Jag1 deletion. Development 137: 4191–4199, 2010. doi:10.1242/dev.052043. 21068062 PMC2990210

[106] Shen D, Li J, Lepore JJ, Anderson TJ, Sinha S, Lin AY, Cheng L, Cohen ED, Roberts JD Jr, Dedhar S, Parmacek MS, Gerszten RE. Aortic aneurysm generation in mice with targeted deletion of integrin-linked kinase in vascular smooth muscle cells. Circ Res 109: 616–628, 2011. doi:10.1161/CIRCRESAHA.110.239343. 21778429 PMC3351207

[107] Zhang H, Gu S, Al-Sabeq B, Wang S, He J, Tam A, Cifelli C, Mathalone N, Tirgari S, Boyd S, Heximer SP. Origin-specific epigenetic program correlates with vascular bed-specific differences in Rgs5 expression. FASEB J 26: 181–191, 2012. doi:10.1096/fj.11-185454. 21965603

[108] Rensen SS, Doevendans PA, van Eys GJ. Regulation and characteristics of vascular smooth muscle cell phenotypic diversity. Neth Heart J 15: 100–108, 2007. doi:10.1007/BF03085963. 17612668 PMC1847757

[109] Owens GK, Kumar MS, Wamhoff BR. Molecular regulation of vascular smooth muscle cell differentiation in development and disease. Physiol Rev 84: 767–801, 2004. doi:10.1152/physrev.00041.2003. 15269336

[110] Beamish JA, He P, Kottke-Marchant K, Marchant RE. Molecular regulation of contractile smooth muscle cell phenotype: implications for vascular tissue engineering. Tissue Eng Part B Rev 16: 467–491, 2010. doi:10.1089/ten.TEB.2009.0630. 20334504 PMC2943591

[111] Toda T, Tsuda N, Takagi T, Nishimori I, Leszczynski D, Kummerow F. Ultrastructure of developing human ductus arteriosus. J Anat 131: 25–37, 1980. 7440403 PMC1233285

[112] Salvador J, Hernandez GE, Ma F, Abrahamson CW, Pellegrini M, Goldman R, Ridge KM, Iruela-Arispe ML. Transcriptional evaluation of the ductus arteriosus at the single-cell level uncovers a requirement for Vim (Vimentin) for complete closure. Arterioscler Thromb Vasc Biol 42: 732–742, 2022. doi:10.1161/ATVBAHA.121.317172. 35443793 PMC9806842

[113] Zou M, Mangum KD, Magin JC, Cao HH, Yarboro MT, Shelton EL, Taylor JM, Reese J, Furey TS, Mack CP. Prdm6 drives ductus arteriosus closure by promoting ductus arteriosus smooth muscle cell identity and contractility. JCI Insight 8: e163454, 2023. doi:10.1172/jci.insight.163454. 36749647 PMC10077476

[114] Gao Q, Zhan P, Alander CB, Kream BE, Hao C, Breyer MD, Pilbeam CC, Raisz LG. Effects of global or targeted deletion of the EP4 receptor on the response of osteoblasts to prostaglandin in vitro and on bone histomorphometry in aged mice. Bone 45: 98–103, 2009. doi:10.1016/j.bone.2009.03.667. 19344793

[115] Moir LM, Ward JP, Hirst SJ. Contractility and phenotype of human bronchiole smooth muscle after prolonged fetal bovine serum exposure. Exp Lung Res 29: 339–359, 2003. doi:10.1080/01902140303758. 12888448

[116] Asai D, Kawano T, Murata M, Nakashima H, Toita R, Kang JH. Effect of fetal bovine serum concentration on lysophosphatidylcholine-mediated proliferation and apoptosis of human aortic smooth muscle cells. J Oleo Sci 69: 255–260, 2020. doi:10.5650/jos.ess19268. 32051357

[117] Lee KJ, Hinek A, Chaturvedi RR, Almeida CL, Honjo O, Koren G, Benson LN. Rapamycin-eluting stents in the arterial duct: experimental observations in the pig model. Circulation 119: 2078–2085, 2009. doi:10.1161/CIRCULATIONAHA.107.737734. 19349326

[118] Li M, Ye L, Ye X, Wang S, Zhang H, Liu J, Hong H. Hypoxia-induced ARHGAP26 deficiency inhibits the proliferation and migration of human ductus arteriosus smooth muscle cell through activating RhoA-ROCK-PTEN pathway. J Cell Biochem 120: 10106–10117, 2019. doi:10.1002/jcb.28294. 30592323

